# Targeting Dual Immune Checkpoints PD‐L1 and HLA‐G by Trispecific T Cell Engager for Treating Heterogeneous Lung Cancer

**DOI:** 10.1002/advs.202309697

**Published:** 2024-09-05

**Authors:** Yu‐Chuan Lin, Mei‐Chih Chen, Shi‐Wei Huang, Yeh Chen, Jennifer Hui‐Chun Ho, Fang‐Yu Lin, Xiao‐Tong Tan, Hung‐Che Chiang, Chiu‐Ching Huang, Chih‑Yen Tu, Der‐Yang Cho, Shao‐Chih Chiu

**Affiliations:** ^1^ Translational Cell Therapy Center China Medical University Hospital No. 2, Yude Rd., North Dist. Taichung City 404 Taiwan; ^2^ Shine‐On BioMedical Co. Ltd. Rm. B, 10F., No. 573, Sec. 2, Taiwan Blvd., West Dist. Taichung City 403 Taiwan; ^3^ Institute of New Drug Development China Medical University Taichung City 404 Taiwan; ^4^ Institute of Biomedical Sciences National Chung Hsing University Taichung City 402 Taiwan; ^5^ Department of Food Science and Biotechnology National Chung Hsing University Taichung City 402 Taiwan; ^6^ Center for Translational Genomics and Regenerative Medicine Research China Medical University Hospital Taichung City 404 Taiwan; ^7^ Department of Ophthalmology China Medical University Hospital China Medical University Taichung City 404 Taiwan; ^8^ Department of Medical Research Eye Center China Medical University Hospital Taichung City 404 Taiwan; ^9^ College of Medicine China Medical University Taichung City 404 Taiwan; ^10^ Division of Nephrology and the Kidney Institute Department of Internal Medicine China Medical University Hospital Taichung City 404 Taiwan; ^11^ Division of Pulmonary and Critical Care Department of Internal Medicine China Medical University Hospital Taichung City 404 Taiwan; ^12^ School of Medicine College of Medicine China Medical University Taichung City 404 Taiwan; ^13^ Drug Development Center China Medical University Taichung City 404 Taiwan; ^14^ Department of Neurosurgery China Medical University Hospital Taichung City 404 Taiwan

**Keywords:** human leukocyte antigen‐G (HLA‐G), immune checkpoint (ICP), nanobody‐based trispecific T cell engager (Nb‐TriTE), non‐small cell lung cancer (NSCLC), programmed death‐ligand 1 (PD‐L1)

## Abstract

Immunotherapy targeting immune checkpoints (ICPs), such as programmed death‐ligand‐1 (PD‐L1), is used as a treatment option for advanced or metastatic non‐small cell lung cancer (NSCLC). However, overall response rate to anti‐PD‐L1 treatment is limited due to antigen heterogeneity and the immune‐suppressive tumor microenvironment. Human leukocyte antigen‐G (HLA‐G), an ICP as well as a neoexpressed tumor‐associated antigen, is previously demonstrated to be a beneficial target in combination with anti‐PD‐L1. In this study, a nanobody‐based trispecific T cell engager (Nb‐TriTE) is developed, capable of simultaneously binding to T cells, macrophages, and cancer cells while redirecting T cells toward tumor cells expressing PD‐L1‐ and/or HLA‐G. Nb‐TriTE shows broad spectrum anti‐tumor effects in vitro by augmenting cytotoxicity mediated by human peripheral blood mononuclear cells (PBMCs). In a humanized immunodeficient murine NSCLC model, Nb‐TriTE exhibits superior anti‐cancer potency compared to monoclonal antibodies and bispecific T cell engagers. Nb‐TriTE, at the dose with pharmacoactivity, does not induce additional enhancement of circulating cytokines secretion from PMBCs. Nb‐TriTE effectively prolongs the survival of mice without obvious adverse events. In conclusion, this study introduces an innovative therapeutic approach to address the challenges of immunotherapy and the tumor microenvironment in NSCLC through utilizing the dual ICP‐targeting Nb‐TriTE.

## Graphical abstract

A nanobody‐based trispecific T cell engager (Nb‐TriTE) is developed to redirect T cells to non‐small cell lung cancer (NSCLC) through dual ICP targeting. In vitro and in vivo evidence suggests that Nb‐TriTE can overcome the immune checkpoints (ICP) heterogeneity of NSCLC without obvious toxicity, implying an innovative approach for the treatment of NSCLC.

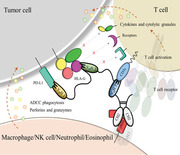

## 1 Introduction

Lung cancer is one of the leading malignancies worldwide and has the highest mortality rate among all cancers.^[^ ^1^ ^]^ Non‐small‐cell lung cancer (NSCLC) represents the primary subtype of lung cancer, with adenocarcinoma serving as its predominant histological manifestation.^[^ ^2^ ^]^ Despite availability of a comprehensive range of treatment options, a significant proportion of NSCLC patients still experience a poor prognosis due to diagnosis of NSCLC at an advanced stage.^[^ ^3^ ^]^

Immunotherapy has witnessed substantial growth in recent years as a treatment modality for cancer. Notably, immune checkpoint blockade (ICB) antibodies targeting the programmed cell death protein‐1 (PD‐1)/programmed death‐ligand 1 (PD‐L1) axis, such as Nivolumab, Pembrolizumab, and Atezolizumab, have demonstrated initial high response rates in various malignancies.^[^ ^4^ ^]^ However, a substantial portion of patients fail to derive long term benefits from this treatment, majority due to the rapid development of drug resistance.^[^ ^5^ ^]^ Likewise, the efficacy of ICB therapy targeting the PD‐1/PD‐L1 axis in the treatment of advanced NSCLC remains unsatisfactory.^[^ ^6^ ^]^

Activation of inhibitory immune checkpoints (ICPs) other than PD‐1/PD‐L1 in tumor microenvironment (TME) during treatment period may affect the efficacy of PD‐1/PD‐L1 antibodies.^[^ ^7^ ^]^ In addition to PD‐1, a variety of inhibitory ICPs on T cells, such as T cells immune globulin mucin‐3 (TIM‐3), cytotoxic T‐lymphocyte‐associated antigen‐4 (CTLA‐4), lymphocyte activation gene 3 (LAG‐3), are associated with T cell function.^[^ ^7^, ^8^ ^]^ Distinct from ICPs on T cells, human leukocyte antigen G (HLA‐G) has been identified as an potent ICP as well as a neo‐expressed tumor‐associated antigen (TAA) in a large proportion of solid tumors.^[^ ^9^ ^]^ Current understanding acknowledges that immune checkpoints work together synergistically to create an immune inhibitory effect. The singular antagonism of the PD‐1/PD‐L1 pathway alone has limited effectiveness in enhancing immune cell function and is prone to drug resistance.^[^ ^5a^ ^]^ Therefore, combining multiple checkpoint inhibitors may be a strategy to overcome the resistance to single ICB therapy. Regarding this strategy, we have demonstrated that dual targeting of PD‐L1 and HLA‐G, in combination with chemotherapy, potentiates the cytotoxicity of T lymphocytes against NSCLC.^[^ ^10^ ^]^

Complex networks in TME may influence the progression of malignancy and the efficacy of immunotherapy. Factors such as the absence of PD‐L1 expression, loss of tumor‐infiltrating lymphocytes (TILs), a decreased CD8^+^ T/Tregs ratio, and the exhaustion of CD8^+^ T cells have been implicated in rendering resistance against PD‐1/PD‐L1 ICB therapy.^[^ ^11^ ^]^ In particular, the lack of significant TILs in tumor regions is the major problem limiting the efficacy of T cell‐based immunotherapies.^[^ ^12^ ^]^ Therefore, strategies aimed at modulating TIL characteristics within the TME and enhancing the efficient interactions between effector cells and tumor cells hold the potential to enhance the efficacy and prognosis of immunotherapy in the treatment of NSCLC.

Bispecific T cell engager (BiTE) is a bispecific antibody construct with unique capabilities that simultaneously binds with one arm to a TAA on tumor cells and with another arm to CD3 complex on T cells. This binding process creates a cytolytic synapse independently of the intrinsic antigen‐specific T cell receptor (TCR) recognition.^[^ ^13^ ^]^ BiTE stands as one of the most promising therapeutic strategies to address the limitation of TILs.^[^ ^11^, ^12^ ^]^ Given the favorable outcomes of BiTE therapy in hematological malignancies, a number of BiTE antibodies directed against TAAs are currently undergoing clinical development.^[^ ^14^ ^]^ Nevertheless, the limited effectiveness of BiTE therapeutics in treating solid tumors, mainly due to the heterogeneity of TAAs, is a major challenge.^[^ ^14b,c^ ^]^ Additionally, the short serum half‐life of BiTE, requiring continuous systemic infusion, is a hurdle to be overcome.^[^ ^15^ ^]^ Moreover, limited ability of BiTE to infiltrate the regions of solid tumors and the immunosuppressive TME within solid tumors pose additional challenges for BiTE therapeutics.^[^ ^16^ ^]^ Therefore, the engineering of antibody constructs with the goal of improving therapeutic efficacy and minimizing toxicity is an emerging topic in cancer therapeutic development to address the limitations of BiTE therapies for the treatment of solid tumors.

Trispecific T cell engagers (TriTEs) are being actively developed for the treatment of solid tumors in the wake of BiTE therapy.^[^ ^17^ ^]^ The binding affinity, epitope position, flexibility, and ability to form immunological synapses are factors that influence the efficiency of multispecific T cell engagers in treating solid tumors.^[^ ^15a^ ^]^ TriTEs can either target an additional TAA via a third binding moiety to increase specificity and avoid immune escape, or target additional costimulatory receptors on T cells to improve their effector functions.^[^ ^13a^ ^]^ Our previous studies have shown that targeting HLA‐G could overcome the immunosuppressive TME and combat drug resistance in solid tumors.^[^ ^18^ ^]^ Furthermore, the dual‐targeting approach against both HLA‐G and PD‐L1 is a promising strategy to overcome tumor heterogeneity and potentiate T cell‐based immunotherapy for the treatment of solid tumors.^[^ ^10^, ^19^ ^]^ In the present study, we developed a nanobody‐based dual ICP‐targeting TriTE (Nb‐TriTE) constructed by fusing anti‐PD‐L1, anti‐HLA‐G, and anti‐CD3 Nbs to a human immunoglobulin G (IgG) crystallizable fragment (Fc) domain and evaluate the potency as well as safety of Nb‐TriTE in the treatment of NSCLC in vitro and in vivo.

## 2 Results

### 2.1 Upregulation and Compensatory Expression of PD‐L1 and HLA‐G in Refractory NSCLC

**Figure 1:**
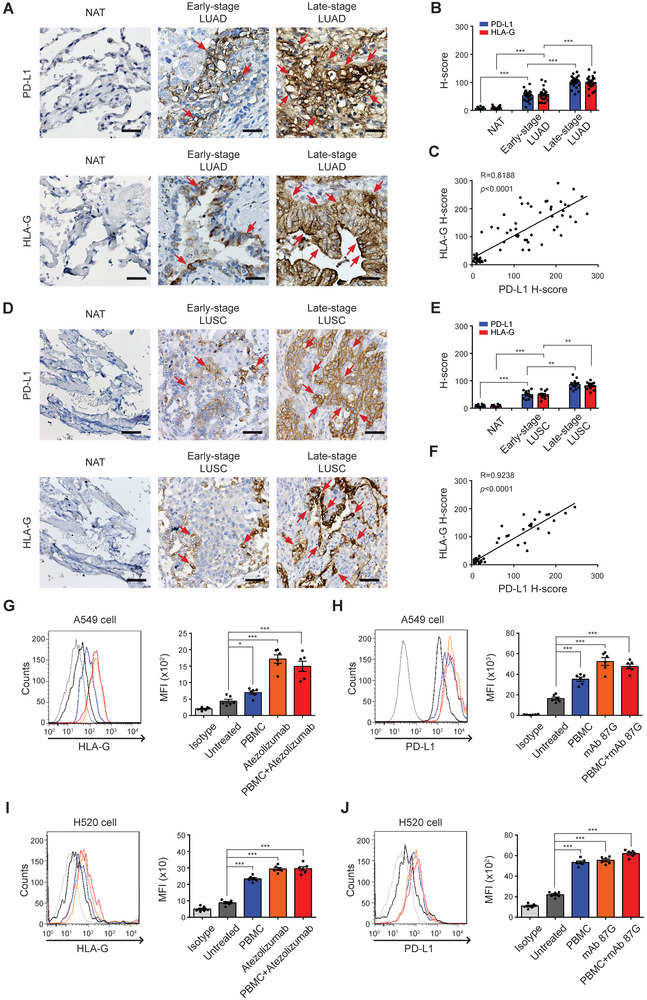
PD‐L1 and HLA‐G are highly expressed in refractory NSCLC. A) Programmed death‐ligand 1 (PD‐L1) and human leukocyte antigen‐G (HLA‐G) staining were performed on human LUAD tissue slices from different stages. Paired normal adjacent noncancerous tissues (NAT) were served as a control. B) Quantitative analysis for PD‐L1 and HLA‐G staining are shown as H‐scores respectively. Data are means ± SEM of 44 independent samples. C) The correlation between PD‐L1 and HLA‐G expression in LUAD was evaluated and quantified using Pearson's correlation coefficient (R). In parallel, D) PD‐L1 and HLA‐G staining were performed on human LUSC tissue slices from different stages. Paired NAT were served as a control. E) Quantitative analysis for PD‐L1 and HLA‐G staining are shown as H‐scores respectively. Data are means ± SEM of 22 independent samples. F) The correlation between PD‐L1 and HLA‐G expression in LUSC was evaluated and quantified using Pearson's correlation coefficient (R). G)The expression of HLA‐G on A549 and I) H520 cell membrane was examined by flow cytometric analysis, following a 24‐h treatment with PBMC, Atezolizumab, or PBMC + Atezolizumab. Quantitative results are shown as mean fluorescence intensity (MFI). Data represent means ± SEM of six independent experiments. H) The expression of PD‐L1 on A549 and J) H520 cell membrane was examined by flow cytometric analysis, following a 24‐h treatment with PBMC, HLA‐G monoclonal antibody clone 87G (mAb 87G), or PBMC + mAb 87G. Quantitative results are shown as MFI. Data represent means ± SEM of six independent experiments. *p < 0.05; *p < 0.01; ***p < 0.001. NSCLC, non‐small cell lung cancer; LUAD, lung adenocarcinoma; LUSC, lung squamous cell carcinoma.

To assess the correlation between disease stage and the expression levels of PD‐L1 and HLA‐G in NSCLC, specimen collection was carried out in accordance with the Institutional Review Board (IRB) guidelines. Subsequently, tumor sections were prepared and subjected to immunohistochemistry (IHC) staining, as detailed in the Experimental Section. The results showed that the expression levels of PD‐L1 and HLA‐G in advanced NSCLC tumors were higher than those in early‐stage tumors, with no detectable expression observed in the paired normal tissue adjacent to the tumor (NAT) (**Figure** **1**A–E). Moreover, further analysis revealed a positive correlation between PD‐L1 and HLA‐G expression levels in patient with adenocarcinoma (LUAD, R = 0.8188, *p* < 0.0001. Figure 1C) and squamous cell carcinoma (LUSC, R = 0.9238, *p* < 0.001. Figure 1F). It is known that the limited efficacy of PD‐1/PD‐L1 ICB therapy may result from the activation of other ICP pathways.^[^ ^7^ ^]^ We therefore hypothesized that the expression profile of HLA‐G on NSCLC tumor cells would be affected following treatment with PD‐L1 blocking antibodies, and vice versa. To verify this, we treated NSCLC cell lines A549 and H520 with the clinical anti‐PD‐L1 monoclonal antibody Atezolizumab alone or in combination with peripheral blood mononuclear cells (PBMCs), and subsequently detected changes in HLA‐G levels. The expression levels of HLA‐G on the surface of NSCLC cells were significantly enhanced after treatment with Atezolizumab or cocultured with PBMCs in the presence of Atezolizumab (Figure 1G,I). Conversely, a significant increase in PD‐L1 expression levels on the surface of NSCLC cells was observed following treatment with a commercial HLA‐G blocking/neutralizing antibody (mAb 87G) or when cocultured with PBMCs in combination with mAb 87G (Figure 1H,J). Therein, the exhausted markers on PMBCs were not affected during the in vitro coculturing (Figure S1, Supporting Information).

### 2.2 Development of Nb‐TriTE for the Dual ICB Targeting HLA‐G and PD‐L1

**Figure 2:**
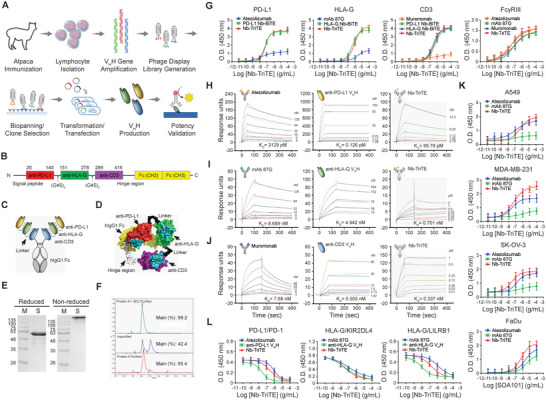
Characterization of Nb‐TriTE. A) Schematic representation for the selection and identification process of V_H_H nanobodies in this study. B) The construction of Nb‐TriTE was engineered with three V_H_H nanobodies specific to anti‐PD‐L1, anti‐HLA‐G, and CD3ɛ, respectively, following a secreatable signal peptide. These three V_H_H fragments were connected through two flexible GS linkers (G4S)_2_ and followed by the human IgG1 Fc regions. C) Schematic diagram showing the components and conformation of the developed Nb‐TriTE. D) The 3D structure of Nb‐TriTE was predicted by the Alphafold2 software. E) Reduced or non‐reduced sodium dodecyl sulphate‐polyacrylamide gel electrophoresis was performed to confirm the molecular weight of Nb‐TriTE. F) The purity of Nb‐TriTE was determined by size exclusion chromatography using the Superdex 200 Increase columns. G) Binding ability of Nb‐TriTE and Nb‐BiTE to specific antigens, including human PD‐L1, HLA‐G, and CD3, and the interaction between the Fc region of Nb‐TriTE and Fc gamma receptor III (FcγRIII) were assessed and compared to that of monoclonal antibodies using ELISA. The antibody‐antigen interactions were analyzed by BIAcore SPR biosensor. Sensor chips were coated with H) rhPD‐L1, I) rhHLA‐G, or J) rhCD3, followed by binding with commercial monoclonal antibodies including Atezolizumab targeting PD‐L1, mAb 87G targeting HLA‐G, and Muromonab targeting CD3, the individual V_H_H components of Nb‐TriTE, or Nb‐TriTE. Results are presented as sensorgrams with the equilibrium dissociation constant (*K_D_*). K) Solid tumor cell lines including A549, MDA‐MB‐231, SK‐OV‐3, and FaDu cells were individually seeded onto 96‐well plates. These cells were then incubated with the commercial antibodies Atezolizumab, mAb 87G, and Nb‐TriTE, respectively. The binding affinity of Atezolizumab, mAb 87G, and Nb‐TriTE toward to these cancer cells was analyzed. L) Nb‐TriTE combined with recombinant human PD‐1, KIR2DL4, LILRB1 proteins, followed by added into 96‐well plates that were pre‐coated with PD‐1 or HLA‐G antigen. The ability of Nb‐TriTE competing the binding of PD‐L1/PD‐1, HLA‐G/KIR2DL4, or HLA‐G/KIR2DL4 was examined. The data represented are the means ± SEM of six independent experiments.

Solely targeting the PD‐1/PD‐L1 pathway has limited effectiveness in boosting immune cell function.^[^ ^5a^ ^]^ Our previous studies have demonstrated the feasibility of the dual ICB strategy.^[^ ^10^ ^]^ In the present study, we further developed a nanobody (V_H_H)‐based trispecific T cell engager (Nb‐TriTE) targeting both PD‐L1 and HLA‐G. Each V_H_H fragment within Nb‐TriTE originates from the camelids after immunization, followed by the construction of a V_H_H library using phage display techniques. After multiple rounds of screening and selection, V_H_H antibody candidates with high affinity were determined and sequenced. Following rigorous affinity testing with their respective antigens, we carefully selected clones to develop the Nb‐TriTE antibody construct (**Figure** **2**A). The engineered Nb‐TriTE comprised the following sequences: anti‐PD‐L1 V_H_H, anti‐HLA‐G V_H_H, and anti‐CD3ε V_H_H (Figure 2B). Two flexible (GGGGS)_2_ linker sequences were introduced between these three V_H_H Nbs, followed by fusion with the human IgG1 hinge and Fc region to prolong the half‐life of Nb‐TriTE in the serum (Figure 2C). The predicted structure constructed using an algorithm derived from the 3D protein prediction database Alphafold2 is shown in Figure 2D. The production of Nb‐TriTE was characteristic through sodium dodecyl sulfate‐polyacrylamide gel electrophoresis (SDS‐PAGE) analysis (Figure 2E) and size‐exclusion high‐performance liquid chromatography after purified from harvested cell culture fluid (Figure 2F). The binding affinities of Nb‐TriTE to its targets, including recombinant human PD‐L1 (rhPD‐L1), rhHLA‐G, and rhCD3ε, as well as the interaction between the Fc region of Nb‐TriTE and Fc gamma receptor III (FcγRIII), were assessed and compared with the commercial antibodies (Atezolizumab: anti PD‐L1, mAb 87G: anti HLA‐G, and Muromonab (OKT3): anti‐CD3) and Nb‐BiTEs through enzyme‐linked immunosorbent assay (ELISA). The results show that Nb‐TriTE and Nb‐BiTEs had comparable binding abilities to their target ligands, and both are superior to the corresponding monoclonal antibodies. (Figure 2G). Additionally, the comparison analysis of binding interactions between commercial monoclonal antibodies, the V_H_H components of Nb‐TriTE, and Nb‐TriTE itself with target antigens, including recombinant rhPD‐L1, rhHLA‐G, and rhCD3ε, was conducted using surface plasmon resonance (SPR) assay on a BIAcore SPR biosensor (BIAcore Inc). The results indicate that the equilibrium dissociation constant (*K_D_*) of Nb‐TriTE (95.76 pm) binding to rhPD‐L1 was higher than that of its anti‐PD‐L1 V_H_H component (0.126 pm), while >32.7‐fold lower than that of monoclonal Ab Atezolizumab (3129 pm) (Figure 2H). The *K_D_* for Nb‐TriTE and anti‐HLA‐G V_H_H binding to rhHLA‐G were 0.701 and 4.942 nm, respectively, both were lower than that of mAb 87G (8.689 nm) (Figure 2I). The *K_D_* values for Nb‐TriTE (0.337 nm) and anti‐CD3ε V_H_H (0.505 nm) binding to rhCD3ε were both lower than that of the commercial monoclonal antibody Muromonab (7.68 nm), demonstrating a better binding affinity and specificity of Nb‐TriTE and its anti‐CD3ε V_H_H component compared to Muromonab (Figure 2J). Subsequently, the recognition and binding ability of Nb‐TriTE to cancer cells was evaluated through a cell‐based ELISA. HLA‐G and/or PD‐L1 expressing cancer cells including NSCLC A549, breast cancer MDA‐MB‐231, ovarian cancer SK‐OV‐3, and hypopharyngeal tumor FaDu cell lines were used. The results demonstrate a greater ability of Nb‐TriTE to recognize cancer cells than commercial antibodies (Figure 2K). Afterward, competitive ELISA was performed to assess and compare the specificity and capacity of Nb‐TriTE with its V_H_H components and commercial monoclonal Abs in inhibiting PD‐L1 binding to PD‐1, HLA‐G binding to KIR2DL4, as well as HLA‐G binding to LILRB1. (Figure 2L). Moreover, the purified Nb‐TriTE and its anti‐HLA‐G V_H_H component showed strong binding affinity to all HLA‐G isoforms, comparable to that of the mAb 4H84 and superior to mAb 87G (Figure S2A–D, Supporting Information). Although Nb‐TriTE can bind all HLA‐G isoforms, it had the capacity to recognize membrane‐bound HLA‐G overexpressed on tumor cells even in the presence of interference from soluble HLA‐G isoforms (Figure S2E,F, Supporting Information).

### 2.3 Nb‐TriTE Simultaneously Binding on Tumor and Immune Cells

**Figure 3:**
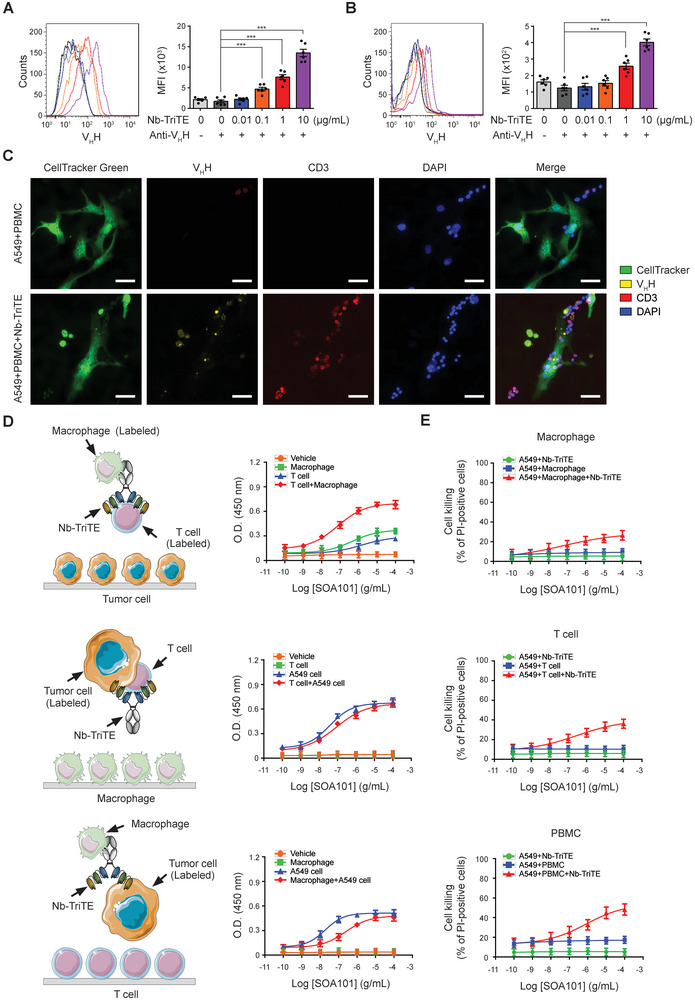
Nb‐TriTE simultaneously connects tumor cells with T cells, further enhancing the cell killing activity of T cells and immune cells in PBMCs. A) A549 cells, with a density of 1 × 10^5^ cells mL^−1^, and B) PBMCs, with a density of 3 × 10^5^ cells mL^−1^ were independently incubated with 0, 0.01, 0.1, 1, or 10 µg mL^−1^ Nb‐TriTE, followed by incubation with anti‐camelid V_H_H‐iFluor 555 Ab. The binding levels of Nb‐TriTE were determined by flow cytometric analysis, and quantitative results are represented as mean fluorescence intensity (MFI). The data presented are the means ± SEM of six independent samples. C) A549 cells were pre‐labeled with CellTracker Green and seeded into 12‐well plates, then followed by incubation with PBMCs in the presence or absence of Nb‐TriTE. Immunocytochemistry was performed to examine the association between A549 cells, CD3^+^ T cells, and Nb‐TriTE using fluorescence microscopy. The original magnification is ×400. The scale bar represents 50 µm. D) The schematic diagrams show the experimental strategy for evaluating the linkage between A549 cells, T cells, macrophages, and Nb‐TriTE through cell‐based ELISA. HRP‐labeled E‐cadherin^+^ A549 cells with HRP‐labeled CD3^+^ T cells, HRP‐labeled E‐cadherin^+^ A549 cells with HRP‐labeled CD68^+^ macrophages, and HRP‐labeled CD3^+^ T cells with HRP‐labeled CD68^+^ macrophages were incubated with Nb‐TriTE at a series concentration and then sequentially introduced into culture plates pre‐seeded with macrophages, T cells, and A549 cells at a density of 1 × 10^5^ cells mL^−1^. After incubation with 3,3′,5,5′‐tetramethylbenzidine, the reaction was terminated using 2N sulphuric acid, and then the absorbance at 450 nm was determined using ELISA reader. E) A549 cells were seeded into 24‐well plates at a density of 4 × 10^4^ cells mL^−1^ and then stained with CellTracker Green. A549 cells were then cocultured with macrophages, T cells, and PBMC, respectively, in the presence of Nb‐TriTE at a range of concentrations for 24 h. Thereafter, the cocultured cells were stained with propidium iodide (2 µg mL^−1^), and the dead cells were detected using flow cytometer. ***p < 0.001.

Based on the results above, purified Nb‐TriTE could effectively bind to PD‐L1 and HLA‐G on tumor cells, as well as CD3 on T cells. We subsequently evaluated the potency of Nb‐TriTE to concurrently engage with both tumor and immune cells through a coculture system. The identification of macrophages and T cells that utilized in the coculture system, and determination of saturated immune cell number (6 × 10^6^) against 6 × 10^3^ A549 cells per well, as well as the experimental design of ELISA‐based functional assay are described in the Experimental Section (also refer to Figure S3, Supporting Information). The interaction of the A549 cells and Nb‐TriTE, and CD3^+^ T cells with Nb‐TriTE was evaluated. The binding of Nb‐TriTE to CellTracker Green‐pre‐stained A549 cells exhibited a dose‐dependent escalation (**Figure** **3**A). Similarly, a dose‐dependent elevation in the binding quantity of Nb‐TriTE to CD3^+^ T cells was observed (Figure 3B). In addition, after coculturing with PBMCs in the presence of Nb‐TriTE, the fluorescent signals from CD3 and V_H_H displayed strong co‐localization with A549 cells, indicating that the presence of Nb‐TriTE facilitated the engagement between CD3^+^ T cells and A549 cells (Figure 3C). ELISA‐based functional assay was subsequently conducted, and the absorbance values at OD_450_ were determined to assess the simultaneously binding of Nb‐TriTE to tumor cells, T cells, and macrophages (Figure 3D). The results obtained from the functional assays demonstrate the capability of Nb‐TriTE to effectively engage immune cells with tumor cells simultaneously. Consequently, we proceeded to evaluate the impact of Nb‐TriTE on the cytotoxicity of macrophages, T cells, and PBMCs against tumor cells. As depicted in Figure 3E, Nb‐TriTE effectively enhanced the cytotoxicity of macrophages, T cells, and PBMCs, with the most pronounced improvement observed in PBMCs against A549 cells.

### 2.4 Nb‐TriTE Enhances the Anti‐Cancer Capacity of PBMCs In Vitro

**Figure 4:**
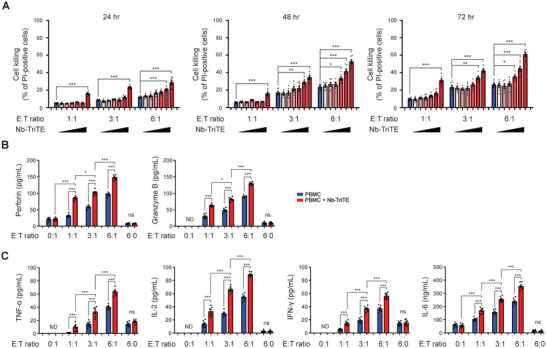
Nb‐TriTE enhances the cell‐killing capacity and cytokine release of PBMCs. A) A549 cells at 4 x 10^4^ were stained with CellTracker Green followed by cocultured with PBMCs at E:T ratios of 1:1, 1:3, and 6:1 in the presence of Nb‐TriTE at concentrations of 0, 0.01, 0.1, 1, 10, and 100 µg mL^−1^, respectively. After coculturing for 24, 48, and 72 h, the cell‐killing ability was evaluated by propidium iodide staining‐based cell death detection and analyzed by flow cytometer. Data represent means ± SEM of six independent samples. A549 cells at 1 × 10^4^ were then incubated with 10 µg mL^−1^ Nb‐TriTE and PBMCs at E:T ratios of 0:1, 1:1, 3:1, 6:1, and 6:0 for 48 h. The release of B) cytotoxic molecules, perforin and granzyme B, and C) cytokines including TNF‐α, IL‐2, IFN‐γ, and IL‐6 in the supernatants were assessed via ELISA. Quantitative results were means ± SEM of six independent samples for each group. *p < 0.05; **p < 0.01; ***p < 0.001. ND, not detected; ns, not significant.

To determine whether the heightened cytotoxicity of PBMCs against tumor cells arises from the engagement facilitated by Nb‐TriTE with tumor cells, T cells and even the macrophages within PBMCs, we conducted an evaluation of in vitro cell killing assay. The evaluation was performed by coculturing of CellTracker Green‐pre‐stained A549 cells with PBMCs at various E:T ratios, both in the presence and absence of Nb‐TriTE. The percentage of dead cells was determined by propidium iodide (PI) staining at 24, 48, and 72 h. The results depicted in **Figure** **4**A illustrate that Nb‐TriTE treatment significantly enhanced the cell‐killing activity of PBMCs in time‐ and dose‐dependent manners. Specifically, at an E:T ratio of 1:1, treatment with 100 µg mL^−1^ Nb‐TriTE demonstrated a significant increase in cell death for A549 cells at 24, 48, and 72 h, in comparison to their respective control groups. At an E:T ratio of 3:1, treatments with 10 and 100 µg mL^−1^ Nb‐TriTE led to a significant enhancement of A549 cell deaths at 48, and 72 h, as compared to their respective control groups. Moreover, at the E:T ratio of 6:1, simultaneous treatments with Nb‐TriTE at concentrations exceeding 1 µg mL^−1^ (1, 10, and 100 µg mL^−1^) exhibited a significant augmentation in PBMC cell killing capacity at 48 and 72 h, in comparison to their control groups.

### 2.5 Nb‐TriTE Enhancement of Cytokines Release from PBMCs in the Presence of Tumor Cells

Subsequently, the release of cytotoxic molecules and cytokines was evaluated via ELISA using the supernatants collected from the coculture groups treated simultaneously with 10 µg mL^−1^ Nb‐TriTE for 48 h. Figure 4B illustrates that in the presence and absence of Nb‐TriTE, an increasing E:T ratio corresponded to an increase in the release of cytotoxic molecules, including perforin and granzyme B, from PBMCs. Notably, Nb‐TriTE treatment significantly potentiated the concentration of perforin and granzyme B in the coculture system in the present of A549 cells. Moreover, cytokines, including TNF‐α, IL‐2, IFN‐γ, and IL‐6, secreted from PBMCs after coculturing with A549 cells were increased in correspondence with the rising E:T ratio, while simultaneous Nb‐TriTE treatment further intensified the secretion of these cytokines (Figure 4C). It is important to note that, in the absence of tumor cells (indicated by an E:T ratio of 6:0), the concentrations of cytotoxic molecules as well as tested cytokines were at minimal levels and not altered by Nb‐TriTE (Figure 4B,C, respectively), supporting that Nb‐TriTE per se has a lower risk of triggering PBMCs to secret cytokine in the absent of tumor cells.

### 2.6 Nb‐TriTE Strengthening Cell‐Killing Capacity of PBMCs Against Tumor Cells Overexpressing PD‐L1 and HLA‐G

**Figure 5:**
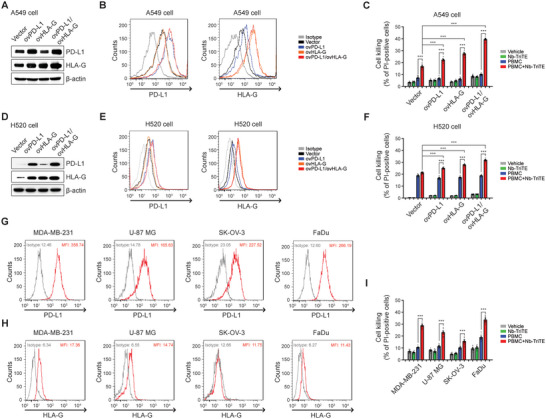
Potent cell‐killing activity of PBMCs in combination with Nb‐TriTE against PD‐L1‐ or HLA‐G‐overexpressing cancer cells. PD‐L1 and HLA‐G levels in mock cells and cells overexpressing PD‐L1 (ovPD‐L1), HLA‐G (ovHLA‐G), and both PD‐L1 and HLA‐G (ovPD‐L1/ovHLA‐G) in A549 cells or H520 cells were determined and compared by A,D) western blot and B,E) flow cytometry analysis using specific antibodies. Flow cytometric data are represented as histogram overlays. C,F) Mock, ovPD‐L1, ovHLA‐G, and ovPDL‐1/ovHLA‐G A549 cells or H520 cells (4 × 10^4^) were stained with CellTracker Green followed by incubation with Nb‐TriTE (10 µg mL^−1^), PBMCs (E:T = 3:1), or PBMCs combined with Nb‐TriTE for 48 h. The cell‐killing ability was then evaluated by PI staining‐based cell death detection via flow cytometer analysis. Data represent the means ± SEM of six independent samples. G) The expression levels of PD‐L1 and H) HLA‐G on cell surface of MDA‐MB‐231, U‐87 MG, SK‐OV‐3, and FaDu cells were detected by flow cytometry. I) Cancer cell lines, including MDA‐MB‐231, U‐87 MG, SK‐OV‐3, and FaDu cells at 4 × 10^4^, were labeled with CellTracker Green followed by incubation with Nb‐TriTE (10 µg mL^−1^), PBMCs (E:T = 3:1), or PBMCs combined with Nb‐TriTE for 48 h. Cell‐killing ability was assessed by propidium iodide staining‐based cell death detection. Data represent the means ± SEM of six independent samples. ***p < 0.001.

The specificity of Nb‐TriTE for PD‐L1 and HLA‐G was further evaluated using A549 subclones that stably overexpress PD‐L1 (ovPD‐L1), HLA‐G (ovHLA‐G), and both PD‐L1 and HLA‐G (ovPD‐L1/ovHLA‐G). The protein levels of PD‐L1 and HLA‐G in A549 sub‐clones were identified by western blotting (**Figure** **5**A) and flow cytometric analysis (Figure 5B). The effect of Nb‐TriTE on anti‐tumor responses of PBMCs was then evaluated and compared between control A549 (represented as vector), ovPD‐L1, ovHLA‐G, and ovPD‐L1/ovHLA‐G A549 sub‐clones. Nb‐TriTE significantly strengthened cell‐killing ability of PBMCs in all the A549 subclones. Among them, the greatest cell‐killing was observed in A549 cells overexpressing both PD‐L1 and HLA‐G (ovPD‐L1/ovHLA‐G) (Figure 5C). The specificity of Nb‐TriTE for PD‐L1 and HLA‐G was further confirmed in H520 cells transiently overexpressing PD‐L1 (ovPD‐L1), HLA‐G (ovHLA‐G), and both PD‐L1 and HLA‐G (ovPD‐L1/ovHLA‐G) as well (Figure 5D‐F).

We conducted additional evaluations of the application of Nb‐TriTE in the treatment of various types of malignant tumors, including the MDA‐MB‐231 triple‐negative breast cancer (TNBC) cell line, U‐87 MG glioblastoma cell line, SK‐OV‐3 ovarian adenocarcinoma cell line, and FaDu hypopharyngeal carcinoma cell line. The expression levels of PD‐L1 and HLA‐G on cell surface of these cell lines were assessed by flow cytometry analysis. The results display the varying levels of PD‐L1 and HLA‐G in theses cell lines (Figure 5G,H, respectively). Cell‐killing was evaluated after co‐culturing individual cell lines with PBMCs in combination with Nb‐TriTE. The results presented in Figure 5I reveal that Nb‐TriTE significantly potentiated cell‐killing capacity of PBMCs for the treatment of all these cancer cell lines. Specifically, Nb‐TriTE exhibited relatively strong enhancement in PBMC cytotoxicity against TNBC cell line MDA‐MB‐231, while it had a comparatively weak effect on cytotoxicity against SK‐OV‐3 cell line in this experiment.

These findings suggest a correlation between the efficacy of Nb‐TriTE in redirecting T cells within PBMCs to eliminate tumor cells and the expression levels of PD‐L1 and HLA‐G on those tumor cells. Moreover, the effectiveness of Nb‐TriTE demonstrates promise in mitigating challenges posed by tumor heterogeneity, particularly variations in PD‐L1 and HLA‐G expression.

### 2.7 Inhibition of Tumor Growth and Survival Prolongation by Nb‐TriTE in a Humanized Orthotopic Lung Cancer Mouse Model

**Figure 6:**
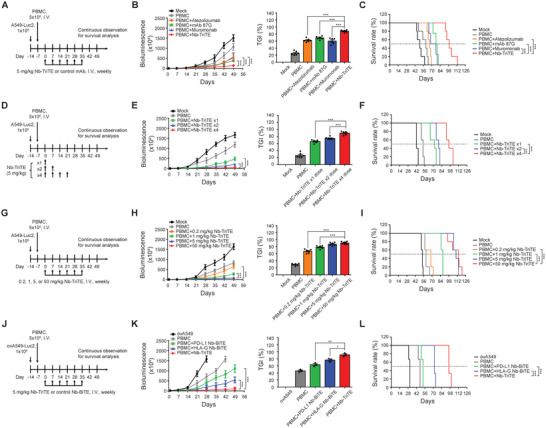
Nb‐TriTE suppresses NSCLC tumor growth and extends survival in humanized xenograft mice. Treatment protocols for animal studies comparing A) the anti‐cancer efficacy of Nb‐TriTE and commercial monoclonal antibodies, D) dosage‐regimen, G) dosage‐ranging, and J) ICP heterogenicity of tumor cells. B,E,H,K) Tumor size was monitored and recorded weekly using the representative in vivo imaging system (IVIS). Tumor changes were quantified as tumor growth inhibition (TGI). C,F,I,L) The survival rate of mice was plotted using the Kaplan–Meier method. Mice were considered dead when bioluminescence reached or exceeded 1.5 × 10^7^. The data is presented as the means ± SEM from five to six independent samples. ovA549, A549 cells overexpressing both PD‐L1 and HLA‐G. *p < 0.05; **p < 0.01; ***p < 0.001.

To further validate the in vivo therapeutic effects of Nb‐TriTE, we conducted experiments using PBMC‐humanized NOD/SCID gamma (NSG) mice bearing cell line‐derived xenografted (CDX) tumors (PBMC‐CDX‐NSG) derived from the NSCLC A549 cell line. In order to compare the efficiency of Nb‐TriTE with commercial monoclonal antibodies, NSCLC tumor‐bearing mice were intravenously injected with PBMCs followed by either Nb‐TriTE or specific monoclonal Ab on a weekly basis for 6 weeks (refer to **Figure** **6**A). The tumor growth was monitored (Figure S4A, Supporting Information) and survival rate was assessed throughout the study. Notably, the tumor growth inhibition (TGI) was significantly higher in the group of PBMCs combined with Nb‐TriTE treatment compared to other groups (Figure 6B). In addition, overall survival (OS) was significantly improved by Nb‐TriTE treatment group in combination with PBMCs compared to PBMCs only (Figure 6C).

### 2.8 Dose‐Dependent Anti‐Cancer Ability of Nb‐TriTE In Vivo

After confirming that Nb‐TriTE had superior in vivo anti‐tumor effects to commercial monoclonal antibodies, we proceeded to assess the efficacy of single‐dose and multiple‐dose Nb‐TriTE treatments in vivo. The experimental protocol for dose regimen comparison experiments is outlined in Figure 6D, and the tumor growth was monitored continuously until day 98 by IVIS (Figure S4B, Supporting Information). The results clearly demonstrate a significant dose‐regimen effect on inhibition of tumor growth following Nb‐TriTE treatment (Figure 6E), which aligns with the observed trends in OS (Figure 6F).

Afterward, dose‐ranging experiments were conducted on a weekly basis over a span of 6 weeks (refer to Figure 6G). Monitoring tumor growth (Figure S4C, Supporting Information) showed a dose‐dependent response in terms of TGI following Nb‐TriTE treatment in conjunction with PBMC (Figure 6H). This response is consistently observed in OS as well (Figure 6I). Moreover, to identify the efficacy of Nb‐TriTE that dual‐targeting PD‐L1 and HLA‐G for the treatment of NSCLC tumors with variations in PD‐L1 and HLA‐G expression, a tumor heterogeneity PBMC‐CDX‐NSG murine model was conducted by inoculating ovPD‐L1/ovHLA‐G A549 cells into mice. Subsequently, Nb‐TriTE, PD‐L1 Nb‐BiTE, and HLA‐G Nb‐BiTE, respectively, were intravenously injected into mice in conjunction with PBMCs once a week for 6 weeks (Figure 6J). The tumor growth was monitored continuously by IVIS (Figure S4D, Supporting Information). Mice treated with Nb‐TriTE combined with PBMCs showed superior TGI and OS outcomes compared to the groups receiving PBMCs alone or PBMCs in conjunction with either PD‐L1 Nb‐BiTE or HLA‐G Nb‐BiTE treatment (Figure 6K,L).

### 2.9 Nb‐TriTE Redirecting T Cells to Tumor Sites Without Evident Toxicities

**Figure 7:**
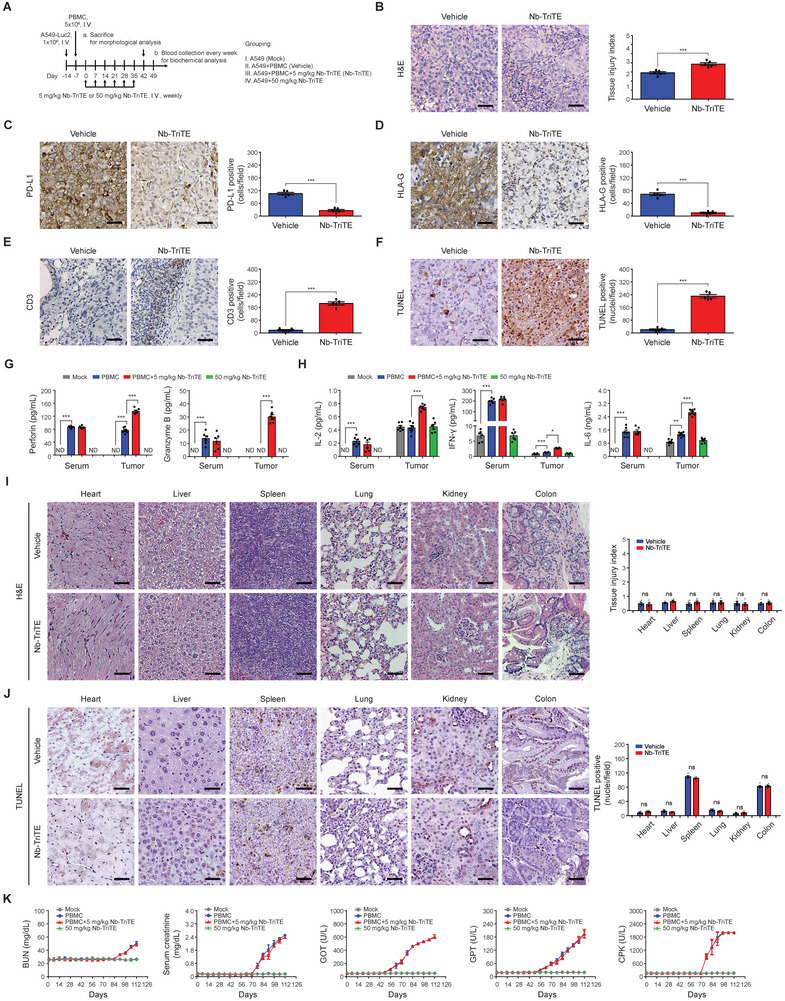
Nb‐TriTE redirects T cells targeting tumor sites without apparent toxicities in humanized NSCLC xenograft mice. A) Treatment protocol for safety experiments. NSG mice were intravenous injected with PMBCs on day −7, followed by intravenous injection with Nb‐TriTE once weekly for six weeks. Mice were sacrificed on day 42 after the initial treatment of Nb‐TriTE. B) H&E staining, and IHC staining for C) PD‐L1, D) HLA‐G, E) CD3, and F) TUNEL staining were performed on the remaining inoculated tumor sections. Mice serum was collected before being sacrificed on day 42. The secretion levels of G) perforin and granzyme B, and H) cytokines including IL‐2, IFN‐γ, and IL‐6 in mice serum and tumor sites were assessed using ELISA. I) H&E stain evaluated the morphological changes in normal tissues and quantified as tissue injury index form six separate samples. J) The apoptotic cells in normal tissues were evaluated by TUNEL staining and shown as positive stained nuclei per field. K) Blood urea nitrogen (BUN), serum creatinine, glutamate‐oxaloacetate transaminase (GOT), glutamate‐pyruvate transaminase (GPT), and creatine‐phospho‐kinase (CPK) were assessed on day 42 after the initial treatment of Nb‐TriTE. The original magnification was ×400. The scale bar in each image represents 50 µm. *p < 0.05; **p < 0.01; ***p < 0.001. ND, not detected; ns, not significant.

An in vivo safety study was performed in the NSG mouse model following the experimental design as described in **Figure** **7**A. Hematoxylin and eosin (H&E) staining was carried out to confirm the characteristics of residual tumors in mice underwent the treatment with or without Nb‐TriTE (Figure 7B). The quantitative results, presented as tissue injury index, reveal significant induction of tissue damage in tumor tissues of the Nb‐TriTE treatment group (*p* < 0.001). IHC staining results suggest that PD‐L1^+^ cells and HLA‐G^+^ cells within tumor burden were significantly reduced after Nb‐TriTE treatment compared with the vehicle group (Figure 7C,D, respectively). In addition to the increase of CD3^+^ cells in the tumor area (Figure 7E), the results of terminal deoxynucleotidyl transferase dUTP nick end labeling (TUNEL) staining indicate a significant increase in apoptotic tumor cells in Nb‐TriTE treatment group (Figure 7F). Moreover, treatment with Nb‐TriTE alone did not induce the secretion of cytolytic molecules, perforin, and granzyme B, in mouse serum and in xenograft tumor area (Figure 7G). Treatment of PBMCs did increase serum perforin and granzyme B. However, the combination of PBMCs with Nb‐TriTE did not lead to further alteration in the serum levels of perforin and granzyme B. Notably, in the tumor region, perforin and granzyme B were significantly increased by Nb‐TriTE combined with PBMCs treatment. The detection results of cytokine release, including IL‐2, INF‐γ, and IL‐6, illustrated in Figure 7H, exhibited a comparable trend. These findings suggest that the presence of Nb‐TriTE may redirect and amplify the anti‐tumor immunity of PBMCs toward the tumor site. Furthermore, it is inferred that the induction of inflammatory cytokines in the serum is solely attributable to PBMCs.

Moreover, the images as well as the quantitative results of H&E staining listed in Figure 7I and TUNEL assay in Figure 7J suggest no obvious injury to vital organs during Nb‐TriTE treatment. Subsequently, examinations of serum biochemistry including blood urea nitrogen (BUN), serum creatinine for kidney function, glutamate‐oxaloacetate transaminase (GOT), glutamate‐pyruvate transaminase (GPT) for liver function, and creatine phosphokinase (CPK) for myocardial damage were performed to evaluate the potential adverse effects after Nb‐TriTE treatment. Weekly testing records for these parameters reveal that the administration of PBMCs leads to the induction of BUN, serum creatinine, GOP, GPT, and CPK after Day 56 (21 days after the last treatment). Notably, co‐treatment with Nb‐TriTE did not amplify or alter the serum concentrations of these factors induced by human PBMCs (Figure 7K). Afterward, tissue cross‐reactivity (TCR) study was conducted to evaluate the reactivity of Nb‐TriTE as well as its V_H_H components to 24 human normal tissues using IHC staining. Nb‐TriTE exhibited minimal reactivity toward most of the normal tissues, with relatively strong binding reactions in placenta, lymph node, thymus, moderate reactions in spleen and colon, and slight reactions in lung, pituitary, and pancreas, which aligns with the pattern of its V_H_H compositions (Figure S5, Supporting Information).

Taken together, these results suggest the successful redirection of T cells to tumor sites by Nb‐TriTE targeting to PD‐L1 and HLA‐G. This redirection resulted in an increased number of CD3^+^ T cells infiltrating to the tumor regions, subsequently leading to the elimination of tumor cells without causing obvious in vivo toxicity.

## 3 Discussion

Immunotherapy has made great progress as a treatment modality for cancer in recent years. Specifically, the emergence of chimeric antigen receptor‐T cell and BiTE therapies has inaugurated a transformative era in the field of cancer treatment. However, addressing the limited availability of antigen targets, the immunosuppressive TME, and the challenges related to penetrating the intricate vasculature of solid tumors remain key hurdles in the treatment of solid tumors.^[^ ^16^, ^18^, ^20^ ^]^ In the present study, we developed a novel approach utilizing an Nb‐based dual ICP‐targeting T cell engager, Nb‐TriTE, consisting of anti‐PD‐L1, anti‐HLA‐G, and anti‐CD3ε V_H_H antibodies (Nbs), to overcome the limitations associated with immunotherapies for the treatment of NSCLC. The in vitro experiments revealed the superior potency of Nb‐TriTE in eliminating NSCLC cells through engaging T cells with tumor cells harboring ICP heterogeneity. The effectiveness of Nb‐TriTE was further corroborated in a humanized mouse model, in which Nb‐TriTE exhibited superior tumor growth inhibition. Specifically, compared with PD‐L1 Nb‐BiTE and HLA‐G Nb‐BiTE, the tumor growth inhibition was superior, and survival was prolonged significantly in mice after treatment with the dual‐targeting Nb‐TriTE in combination with PMBCs. The in vivo experiments indicate the potent efficacy of Nb‐TriTE without evident toxicities.

The intricate networks operating within the TME have a crucial impact on immunotherapy. Recognizing the presence and collaboration of multiple ICPs and the challenges posed by adaptive resistance when solely targeting the PD‐1/PD‐L1 pathway,^[^ ^7a,b^ ^]^ we developed Nb‐TriTE with a novel construct containing V_H_H fragments directed toward PD‐L1 and HLA‐G on tumor cells. This innovative approach is rooted in our prior research, which highlighted the significant benefits of dual targeting these ICPs for enhancing T cell‐based immunotherapy.^[^ ^10^, ^19^ ^]^ Our pioneering Nb‐TriTE marks a significant advancement, comprising V_H_H fragments specific to anti‐PD‐L1, anti‐HLA‐G, and anti‐CD3ɛ. This construct possesses dual ICP targeting characteristics, allowing it to address the ICP heterogeneity within tumors. Moreover, the binding affinity of Nb‐TriTE and individual V_H_Hs within Nb‐TriTE for recombinant human antigens was superior to that of the commercial monoclonal antibodies (Figure 2H–J). Remarkably, Nb‐TriTE exhibits the ability to recognize all HLA‐G isoforms (Figure S2A–D, Supporting Information) and retains the capacity to bind HLA‐G expressed on tumor cells despite interference from soluble HLA‐G isoforms (Figure S2E,F, Supporting Information). Given the capacity of all HLA‐G isoforms to regulate the immune system,^[^ ^21^ ^]^ blocking all HLA‐G isoforms is beneficial for completely disrupting the role of HLA‐G in tumor progression.

In addition to ICP heterogeneity, the insufficient presence of TILs within tumor areas remains a major hurdle for T cell‐based immunotherapies.^[^ ^11c,d^ ^]^ Thus, strategies to enhance TIL characteristics and optimize interactions between effector cells and tumor cells are expected to enhance efficacy and improve NSCLC immunotherapy outcomes. TriTEs outperform BiTEs by targeting additional TAA receptors through a third binding moiety, thereby enhancing specificity, and minimizing the risk of immune escape, or alternatively targeting additional costimulatory receptors on T cells for bolstering the effector function of T cells.^[^ ^13a^ ^]^ In this study, despite the binding affinity of Nb‐TriTE to its ligands being comparable to the corresponding Nb‐BiTEs (Figure 2G), the outcomes in mice treated with Nb‐TriTE and PBMC was superior to those receiving either PD‐L1 Nb‐BiTE or HLA‐G Nb‐BiTE with PBMC. This suggests the effectiveness of Nb‐TriTE with dual‐targeting of PD‐L1 and HLA‐G, overcoming ICP heterogeneity (Figure 6K,L). In addition, the recruitment of CD3^+^ was found in the tumor area (Figure 7E) after Nb‐TriTE treatment, in which the proportions of CD3^+^/TIM3^+^ and CD3^+^/PD‐1^+^ cells were lower compared to the vehicle group (Figure S6, Supporting Information). This suggests that the Nb‐TriTE may effectively redirect and enhance the recruitment of TILs to the tumor area and maintain their activity in the TME.

Furthermore, functional assays demonstrate that Nb‐TriTE could facilitate the simultaneous binding of tumor cells, T cells, and macrophages (Figure 3D), and enhance the killing of tumor cells mediated by T cells, macrophages, and PBMCs (Figure 3E). Additionally, in the in vivo experiments, increases in CD68^+^/CD80^+^ and CD68^+^/CD86^+^ cells around the tumor region imply that Nb‐TriTE attracts or activates macrophages (particularly M1) or dendritic cells (Figure S7A, Supporting Information). These cells are capable of presenting antigens and providing necessary co‐stimulatory signals to T cells.^[^ ^22^ ^]^ Moreover, decreases in CD68^+^/CD163^+^ and CD68^+^/CD206^+^ cells after Nb‐TriTE treatment may imply a reduction in the population of immunosuppressive M2 macrophages within the TME (Figure S7B, Supporting Information). These findings suggest that Nb‐TriTE not only redirects T cells toward tumor cells but also promotes the formation of immune synapse to initiate antibody‐dependent cellular cytotoxicity responses by macrophages or other effector cells. The results showcasing the simultaneous binding of tumor cells to T cells are also in line with in vivo observations of T cell infiltration into tumor regions in the Nb‐TriTE treatment group (Figure 7E). The fusion with human IgG1 Fc (hinge‐CH2‐CH3) suggests extending the plasma half‐life of Nb‐TriTE,^[^ ^23^ ^]^ potentially reducing the need for frequent reinfusions. In the present study, the fusion with human IgG1 Fc in Nb‐TriTE enhanced the secretion of GM‐CSF, MCP‐1, eotaxin, and pro‐inflammatory cytokines, while reducing immunosuppressive cytokine IL‐10 secreting from PBMCs when challenged with tumor cells (Figure S8, Supporting Information). This implies the enhancement of active immune surveillance and anti‐tumor responses. Likewise, the compact size of Nb‐TriTE offers a distinct advantage in penetrating the barriers of solid tumors.

The immune‐mediated toxicities associated with treatment with T cell engagers are a significant challenge that requires attention. Binding of target antigen triggers T cell expansion and proinflammatory cytokine release, recruiting immune cells like macrophages and intensifying the immune response.^[^ ^24^ ^]^ However, this heightened immune response can lead to cytokine release syndrome. Additionally, when applying T cell engagers to treat solid tumors, on‐target/off‐tumor and off‐target/off‐tumor toxicity should be considered.^[^ ^25^ ^]^ In this study, the administration of Nb‐TriTE in the absence of tumor cells does not trigger perforin and granzyme B secretion (Figure 4B) or increase cytokines release (Figure 4C) from human PBMCs in vitro. In the humanized NSG mouse model, Nb‐TriTE enhancement of cytolytic molecules and cytokines released from PBMCs was concentrated at the tumor sites, with no additional amplification of these molecules in mice serum compared with the PBMCs‐treated group (Figure 7G). Furthermore, there were no indications of tissue injury, myocardial damage, liver toxicity, or kidney toxicity in the humanized mouse model after treatment with Nb‐TriTE at the dosage with pharmacoactivity (Figure 7I‐K). These observations, coupled with the results of TCR assay presented in Figure S5 (Supporting Information), suggest a low safety concern regarding the use of Nb‐TriTE in humans.

## 4 Conclusion

In conclusion, nanobody‐based trispecific T cell engager, Nb‐TriTE, targeting both PD‐L1 and HLA‐G on tumor cells along with CD3ɛ on T cells, offers a promising solution to the shortcomings of monospecific ICP antibodies. It provides insight into further combinatorial strategies for immunotherapy against solid tumors, particularly those with elevated PD‐L1 and HLA‐G expression.

## 5 Experimental Section

### Patient Specimens

The specimen collection and slice analysis of NSCLC patients in this study were approved by the IRB of China Medical University Hospital (CMUH), Taichung City, Taiwan (CMUH108‐REC3‐041, CMUH111‐REC3‐233, and CMUH112‐REC3‐186). Patient data pertaining to LUAD and LUSC, encompassing factors such as age, sex, clinical staging, metastasis, progression‐free survival, and overall survival, was obtained from their medical records. H&E staining, followed by microscopic analysis, was performed to thoroughly evaluate the characteristics of LUAD and LUSC.

Forty‐four LUAD patients, comprising 19 women and 25 men, and 22 LUSC patients, comprising 9 women and 13 men, underwent surgery at CMUH, Taiwan. Specimen collection and data acquisition were conducted during these surgeries. In parallel, NAT was systematically collected for use as a control. Following the classification of malignant tumors, the patients were categorized into the following stages: stages I (*n* = 5), stage II (*n* = 17), stage III (*n* = 9), and stage IV (*n* = 13) for LUAD, and stages I (*n* = 6), stage II (*n* = 4), stage III (*n* = 5), and stage IV (*n* = 7) for LUSC. Samples from stages I and II were amalgamated and designated as “early stage”, while samples from stages III and IV were grouped as the “late stage” category.

In addition to H&E staining, PD‐L1 and HLA‐G IHCstaining was performed using specific antibodies against PD‐L1 and HLA‐G, respectively. The H‐scorewas calculated by two pathologists based on both the intensity of staining (graded as follows: 0 for non‐staining, 1 for weak, 2 for median, or 3 for strong, using adjacent normal mucosa as the median) and the proportion of positively stained cells. The possible scores ranged from 0 to 300.^[^ ^26^ ^]^

### Construction of an Expression Plasmid Encoding Nb‐TriTE

The selection and generation of PD‐L1, HLA‐G, and CD3ε‐specific Nbs were executed by Creative Biolabs (NY, USA) using phage display method through the HuSdL human single‐domain antibody library. Briefly, full‐length DNA sequence of human PD‐L1 (NM_001267706.2), HLA‐G (NM_001363567.2), and CD3 (NM_001363567.2) obtaining from NCBI Nucleotide database were converting to amino acid sequences, respectively. The recombinant human PD‐L1, HLA‐G, and CD3ε proteins were then produced to be antigens by HEK293 cells. In parallel, helper phage was prepared by infecting log‐phase E. coli TG1 cells with M13KO7 helper phage for 30 min at 37 °C and then smeared onto 2xTY agar. Plaque was inoculated into 2xTY medium with 50 µg mL^−1^ kanamycin. Supernatants were collected, and the titter was then determined. Subsequently, four rounds of biopanning screening were performed. In brief, PD‐L1, HLA‐G, or CD3 at 10 µg mL^−1^ was coated onto 96‐well plates. After blocking, phage particles at 1 × 10^11^ from phage library were added and incubated for 2 h at 37 °C. Phages were then eluted and neutralized, and the titration was determined. Phage amplification and purification was performed for repeat screening (four rounds). Hundreds of clones were obtained and subjected to phage ELISA for characterization. Candidate phages were inoculated at 2–5 µL well^−1^ into 96‐well plates with 2xYT medium, let grow to exponential phase at 37 °C. In the next day, 50 µL M13KO7 helper phage at 1 × 10^11^ plaque forming unit was added into medium containing bacteria for 16–18 h at 30 °C. The ratio of phage to bacteria was ≈20:1. Binding affinity between the target of interest (PD‐L1, HLA‐G, or CD3) and candidate phages was performed. After coating, blocking, and incubated with candidate phages, 100 µL well^−1^ anti‐M13‐HRP was added for 1 h. TMB substrate was then added for *color* development, and H_2_SO_4_ was used for determining the reaction. Read the absorbance of each well at 450 nm in a microplate reader. Subsequently, synthetic DNA sequences were employed in the construction of the Nb‐TriTE construct. The order of the three Nbs (anti‐PD‐L1, anti‐HLA‐G, and anti‐CD3 V_H_H) in the Nb‐TriTE construct was determined using AlphaFold 2 to predict the 3D structures of different configurations. This ensured that the antigen‐binding regions of each V_H_H component were exposed and capable of perfect binding to their respective epitopes. This construct comprises a signal peptide sequence, followed by anti‐PD‐L1, repeated GGGGS linker, anti‐HLA‐G, repeated GGGGS linker followed by anti‐CD3ε, human IgG1 hinge, and Fc regions. This construct was synthesized using GenBrick synthesis service and subsequently cloned into pcDNA3.1 (Plasmid #138 209, Addgene, Massachusetts, USA) using the XbaI and BamHI restriction sites. The size of the DNA fragments was determined by agarose gel electrophoresis. SPR assays were conducted to analyze the binding affinity of the Nb‐TriTE to the antigens and FcγRIII as well.

### SPR Biosensor Analysis for Nb Binding Affinity

SPR biosensor analysis was conducted on a BIAcore T200 platform with CM5 series sensor chips or protein A chips (GE Healthcare, Illinois, USA) to determine Nb‐TriTE binding affinity. All experiments were performed in degassed PBS (pH 7.4) containing 0.005% Tween‐20 at 25 °C. Briefly, PD‐L1 (Sino Biological, Beijing, China), HLA‐G (Creative BioMart, NY, USA), or CD3ε (Creative BioMart, NY, USA) recombinant proteins were diluted to 20 µg mL^−1^ and immobilized on chips by amine coupling at the flow rate of 5 µL min^−1^ for 7 min (pH 4.5) according to the manufacturer's manual. Commercial antibodies, Nbs, or Nb‐TriTE in HBS‐EP running buffer (GE Healthcare) were then allowed to flow over the activated surface at the same flow rate. Afterward, HBS‐EP running buffer was applied for additional 7 min. The chip was regenerated using 100 mmol L^−1^ glycine and 0.5 mol L^−1^ NaCl (pH 2.0). The association and dissociation constants were monitored using the BiaEval 4.1 software. Kinetic constants were used to determine the *K_D_* values.

### Multiplex Immunoassay for PMBC Cytokine Production

To evaluate the effects of incorporating human IgG1 Fc in Nb‐TriTE on PBMC cytokine production, PBMCs combined with A549 cells at an E:T ratio of 3:1 were incubated with 10 µg mL^−1^ Nb‐TriTE‐Fc or Nb‐TriTE‐8His for 24 h at 37 °C. Nb‐TriTE‐8His that features eight repeated histidine residues, served as a control to the IgG1 Fc hinge. At the indicated time, supernatants from cell cultures were collected, followed by four times dilution. Samples were then added onto 96‐well filter plate for Bio‐Plex Pro human cytokine assay (#M500KCAF0Y, Bio‐Rad). In brief, the filter plate was pre‐wetted, and the assay buffer was removed. Diluted standards and samples were added to a 96‐well filter plate, followed by incubation with 50 µL of antibody beads and 50 µL of AssayChex beads for 2 h at 25 °C, and then at 4 °C for 16 h. After removing the supernatant and washing three times, streptavidin‐PE was added for 40 min at 25 °C. Samples in the filter plate were incubated with reading buffer, and the values were read using a Bio‐Plex 200 system (Bio‐Rad). The output CSV file was used to extract data from Bio‐Plex Data Pro software (#12 015 390, Bio‐Rad). The expression levels of GM‐CSF, MCP‐1, eotaxin, IL‐2, IL‐4, IL‐6, IL‐12, IL‐1β, TNF‐α, IFN‐γ, and IL‐10 were determined.

### Cell Lines and Culture

Human PBMCs were purchased from STEMCELL Technologies (Vancouver, BC, Canada). PBMCs were cultured in X‐VIVO15 medium (Lonza) supplemented with 10% human AB serum (Gemini Bioproducts) and 1000 IU mL^−1^ recombinant human IL‐2 (PeproTech). The human LUAD cell line A549‐luc (American Type Culture Collection, ATCC), LUSC cell line H520 (ATCC), and the human triple‐negative breast cancer cell line MDA‐MB‐231‐luc (MyBioSource, San Diego, United States) were cultured in RPMI‐1640 medium (Thermo Fisher Scientific, Massachusetts, USA). The human ovarian cancer cell line SK‐OV‐3‐Red‐FLuc (PerkinElmer) and the human glioblastoma cell line U‐87 MG‐Luc2 (ATCC) were cultured in McCoy's 5A medium (Sigma‐Aldrich, Massachusetts, USA) and DMEM (Thermo Fisher Scientific), respectively. The oral squamous carcinoma cell line FaDu‐luc2 cells (ATCC) were cultured in EMEM (Thermo Fisher Scientific).

### Cell‐Based ELISA for the Detection of Tumor‐Specific Antibodies

Cell‐based ELISA was performed to evaluate the affinity of commercial antibodies or Nb‐TriTE for target cells. Briefly, A549, MDA‐MB‐231, SK‐OV‐3, and FaDu cells were seeded or coated onto 96‐well plates at a density of 1 × 10^5^ cells mL^−1^ for 24 h. After washing, commercial antibodies, Atezolizumab (Roche, Basel, Switzerland), anti‐human HLA‐G‐neutralized antibody (clone 87G; BioLegend, California, USA.), or Nb‐TriTE were added to the wells and incubated for 1 h at 25 °C. After washing, HRP‐conjugated antibody was introduced into the wells and then incubated for 30 min at 25 °C. Following another round of washing and incubation with 3,3′,5,5′‐tetramethylbenzidine (Agilent, CA, USA) for 15 min, the reaction was terminated using 2N sulphuric acid. Thereafter, the absorbance at 450 nm was determined using a multimode ELISA plate reader (BioTek Instruments, Vermont, USA).

### ELISA‐Based Functional Assay

ImmunoCult human CD3/CD28 T cell activator (STEMCELL) was used to expand T cells from PBMCs. Macrophages were enriched from T cells‐depleted PBMCs using CD3 depletion kit (STEMCELL). The saturated immune cell number toward to 6 × 10^3^ of A549 cells was determined by coculturing respectively with premixed 6 × 10^5^ or 6 × 10^6^ cells of T cells + Nb‐TriTE, macrophages + Nb‐TriTE, or T cells + macrophages + Nb‐TriTE, followed by hybridization with primary antibodies, anti‐CD3 (Novus Biologicals), anti‐CD68 (NeoBiotechnologies), and HRP‐conjugated secondary antibodies. After the optical density of each well was measured by a microplate reader set to 450 nm, the saturated immune cell number against A549 cells (1000:1) was determined. ELISA‐based functional assay was then utilized to verify the simultaneously binding ability of Nb‐TriTE to tumor cells and immune cells. Macrophages, T cells, and tumor cells were coated on the wells of ELISA plates respectively. A series of concentrations of Nb‐TriTE were pre‐incubated with the cell mixture then loaded into ELISA plates as follows: A549 cells and T cells to macrophage‐coated plate, A549 cells and macrophages to T cell‐coated plate, and T cells and macrophages to A549 cell‐coated plate. Primary antibodies against CD3, CD68, and E‐cadherin (GeneTex), which sequentially recognize T cell, macrophage, and A549 cells were added into the plates followed by application of the appropriate HRP‐conjugated secondary antibodies. The absorbance value was then measured at OD 450 nm.

### Cell Killing Assay

Cell‐killing ability was assessed using CellTracker Green (Thermo Fisher Scientific) and PI (Sigma–Aldrich) staining in different groups: tumor cells only, tumor cells + Nb‐TriTE, tumor cells + PBMC, and tumor cells +PBMC + Nb‐TriTE. Briefly, tumor cells were seeded into 24‐well Corning plates at a density of 4 × 10^4^ cells mL^−1^ overnight. The cells were washed and pre‐stained with 1 µg mL^−1^ CellTracker Green for 30 min. Subsequently, the stained tumor cells were cocultured with Nb‐TriTE, PBMC, PBMC + Nb‐TriTE, or culture medium alone. The ratio of effector to target (E:T) was set at 1:1, 3:1, and 6:1. Nb‐TriTE was added at concentrations of 0, 0.01, 0.1, 1, 10, or 100 µg mL^−1^ into 1 mL of medium, and the cells were incubated at 37 °C for 24, 48, and 72 h. The tumor cells‐only group contained only of target cells. Cocultured cells were stained with PI (2 µg mL^−1^) to detect dead cells. Target cells were determined based on their size and CellTracker green staining, while the PI‐labeled populations were considered dead cells. Specific lysis of target cells was determined using the Cytomics FC500 flow cytometer (Beckman Coulter, CA, USA).

### IHC

Tissue sections were processed by antigen retrieval in citrate sodium buffer (Sigma–Aldrich), followed by incubation with H_2_O_2_ for 20 min. After washing with PBS, the sections were blocked with 2% BSA (Sigma–Aldrich) for 30 min and incubated with monoclonal mouse anti‐PD‐L1 antibody (clone ABM4E54; Abcam, Cambridge, UK), mouse anti‐HLA‐G antibody (clone 4H84; Santa Cruz Biotechnology, TX, USA), or rabbit anti‐CD3 antibody (clone SP7, Abcam). The sections were then incubated with HRP‐conjugated secondary anti‐mouse or anti‐rabbit IgG antibodies (Abcam) for 1 h at 25 °C. For IHC, the sections were visualized using 3,3′‐diaminobenzidine (Dako, Glostrup Municipality, Denmark) in accordance with the manufacturer's instructions and counterstained with hematoxylin. Quantification of positively stained cells was performed by two pathologists using Nikon Eclipse 80i upright fluorescent microscope (Nikon, Tokyo, Japan) at ×400 magnification and presented as positive cells per field as previously described.^[^ ^27^ ^]^

### Immunofluorescence

Tissue sections were deparaffinized, rehydrated, and underwent antigen retrieval followed by incubated with 3% hydrogen peroxide for 30 min. After incubation with primary antibodies at 4 °C for 16 h and consequently incubation with appropriate secondary antibodies at 25 °C for 20 min, tissue slides were mounted with ProLong Gold antifade mountant with DAPI (Thermo Fisher Scientific) and photographed with a confocal microscope (Leica TCS SP8 X, Wetzlar, Hesse, Germany). Positive cells were counted at ×400 magnification.

The primary antibodies used in this study are listed below. Alexa Fluor 488 anti‐CD68 (clone KP1, Abcam), anti‐CD80 (clone EPR22183, Abcam), anti‐CD86 (clone EPR21962, Abcam), Alexa Fluor 594 anti‐CD163 (clone EPR19518, Abcam), and Alexa Fluor 555 anti‐mannose receptor (clone EPR22489‐7, Abcam), FITC‐conjugated CD3 (clone UCHT1, Thermo Fisher Scientific), anti‐TIM 3 (clone EPR22241, Abcam), and anti‐PD1 (clone CAL20, Abcam). The secondary antibodies included Alexa Fluor 594‐conjugated goat anti‐rabbit IgG H&L (Abcam) and Alexa Fluor 647‐conjugated goat anti‐rabbit IgG H&L (Abcam).

### Binding Affinity of Anti‐HLA‐G VHH to HLA‐G Isoforms

Full‐length HLA‐G protein containing β2M was purchased from Creative BioMart (Shirley, NY, USA). DNA sequences encoding HLA‐G isoforms were synthesized, amplified, and subcloned into the pcDNA3.4 vector (GenScript). Supernatants from transfected HEK293 cells were collected and purified to obtain HLA‐G isoforms. The molecular weights of HLA‐G isoforms were determined using Coomassie blue staining. The binding abilities of anti‐HLA‐G V_H_H, Nb‐TriTE, and commercial HLA‐G antibodies (mAb 4H84, mAb 87G, and mAb MEM‐G/2) to HLA‐G isoforms were determined by ELISA. A549 cells overexpressing HLA‐G (ovHLA‐G A549 cells) were used for cell‐based ELISA to examine the competitive effects of HLA‐G isoforms on Nb‐TriTE. Different concentrations of soluble HLA‐G isoforms (sHLA‐G5, sHLA‐G6, sHLA‐G7, and their mixture) with constant amounts of Nb‐TriTE were added to 96‐well plates pre‐coated with ovHLA‐G A549 cells. After incubation with HRP‐conjugated anti‐human IgG1 Fc antibodies, the absorbance was measured at OD 450 nm.

### Flow Cytometry

The expression of PD‐L1 and HLA‐G on the cell membranes of A549, ovPD‐L1 A549, or ovHLA‐GA549 cells were examined by flow cytometric analysis. Cells were incubated with or without PBMC, monoclonal anti‐HLA‐G antibody clone 87G (mAb 87G), PBMC + mAb 87G, Atezolizumab, or PBMC + Atezolizumab for 24 h at 37 °C. After trypsinization, the cells were incubated with APC‐conjugated anti‐PD‐L1 Ab (clone MIH1; Thermo Fisher Scientific) or APC‐conjugated anti‐HLA‐G Ab (clone MEM‐G/9, Abcore) for 1 h at 4 °C. Isotypes were used as the staining controls. The expression levels of PD‐L1 and HLA‐G were determined by a flow cytometry and represented as the MFI. In parallel, the proportions of PBMCs expressing TIM‐3, LAG‐3, and TIGIT on cell surface were determined by flow cytometric analysis after cocultured with A549 or H520 cells in the presence of Atezolizumab or mAb 87G for 24 h at 37 °C.

In addition, the binding ability of A549 or CD3^+^ T cells to Nb‐TriTE was evaluated using flow cytometric analysis. Briefly, A549 cells were pre‐stained with CellTracker Green, while PBMCs were incubated with APC‐conjugated anti‐CD3 monoclonal Ab (clone OKT3, Thermo Fisher Scientific). After washing, A549 cells were counted to a density of 1 × 10^5^ cells mL^−1^, and PBMCs were counted to a density of 3 × 10^5^ cells mL^−1^ and incubated with 0, 0.01, 0.1, 1, or 10 µg mL^−1^ Nb‐TriTE together for 1 h at 37 °C. An anti‐camelid V_H_H‐iFluor 555 Ab (GenScript, New Jersey, USA) was added and incubated for 1 h at 4 °C. The expression levels of Nb‐TriTE were determined by flow cytometric analysis and represented as MFI.

### Experimental Animals and Related Ethical Statement

Six to eight weeks old, humanized NSG mice (male, 18 to 20 g) were purchased from the National Laboratory Animal Center and housed at the Animal Center of CMUH (Taichung, Taiwan) to establish the xenograft lung tumor model. Mice were kept in individually ventilated cages and kept at 25 °C with 60% to 70% humidity on a 12‐h light/dark cycle under specific pathogen‐free conditions. Animal care and experimental procedures were conducted under the guidance of the Animal Protection Association of the Republic of China. Minimization of experimental animal numbers and experimental protocols were approved by the Institutional Animal Care and Utilization Committee of China Medical University (CMUIACUC‐2021‐045, CMUIACUC‐2023‐092, and CMUIACUC‐2024‐030) and reviewed by the ethical committee of the Ministry of Science and Technology /National Science and Technology Council, Taiwan (MOST‐110‐2314‐B‐039‐052 and NSCT‐112‐2314‐B‐039‐053).

### Establishment of Xenograft NSCLC Mouse Model for Nb‐TriTE Treatment

Male NSG mice were randomly assigned to control and treatment groups. After anesthetized by intraperitoneal injection of Zoletil (40 mg kg^−1^, Virbac), A549‐luc cells were resuspended in Matrigel (1 × 10^6^ cells/20 µL), and then rapidly percutaneous injected into the right anterior axillary sixth intercostal space at a depth of 5 mm, to establish an orthotopic lung tumor model. Seven days after cell implantation, the mice were infused intravenously with or without 5 × 10^6^ PBMCs/100 µL per mouse (set as day −7). In the Nb‐TriTE treatment groups, 5 mg kg^−1^ Nb‐TriTE was injected via the tail vein on days 0, 7, 14, 21, 28, and 35. Tumor growth was monitored weekly using a novel patented optical IVIS spectrum (PerkinElmer, Massachusetts, USA) with a bioluminescence channel. The mice were sacrificed when the bioluminescence reached 1.5 × 10^7^ or when they experienced severe aphagosis.

Tissue injury index was used to evaluate the safety of Nb‐TriTE treatment based on H&E staining. Injuries are generally characterized by diffuse reactions in the interstitial areas, including neutrophil infiltration, apoptosis, necrosis, fatty changes, cytoplasmic vacuoles, hemorrhage, atrophy, and abnormal cell swelling. Higher scores represent more severe tissue damage in the scoring system: 0, no observed histopathological changes; 1, < 10%; 2, 10–25%; 3, 25–50%; 4, 50–75%; 5, 75–100%.

### Statistical Analyses

All experiments were performed at least in triplicate, and all assay conditions were tested in duplicate. In this study, data were presented as mean ± SEM and analyzed using the Student's t‐test and paired t‐test. For animal experiments, data were presented as mean ± SEM and analyzed using one‐way ANOVA (Analysis of Variance) for comparing means across multiple groups. The survival rate was analyzed using the Kaplan–Meier estimate and log‐rank test. Statistical significance was set at *p* value < 0.05.

### Copyright Statement

Some of the graphic design material pictures used in Figures 2A and S3D (Supporting Information) were recreated using BioRender.com assets. Selected artwork shown in Figure 2A, Figure 3D, Figure S3C,D (Supporting Information) were adapted from images provided by Servier Medical Art (https://smart.servier.com) and is licensed under a Creative Commons Attribution 4.0 Unported License.

## Conflict of Interest

Y.C.L., J.H.C.H., and H.C.C. are employed by Shine‐On BioMedical Co., Ltd. during the conduct of this study. C.C.H. and S.C.C. are employed and owned equity interests in Shine‐On BioMedical Co., Ltd. during the conduct of this study. The other authors declare no direct conflicts of interest.

## Author Contributions

Y.C.L. and M.C.C. contributed equally to this work. S.C.C. and D.Y.C. correspond equally to this study. Y.C.L., S.C.C., and D.Y.C. designed the study; Y.C.L., S.W.H., Y.C., F.Y.L., and X.T.T. conducted the experiments; Y.C.L., S.W.H., Y.C., J.H.C.H., and S.C.C. acquired data; Y.C.L., M.C.C., H.C.C. C.Y.T., and C.C.H. analyzed data; Y.C.L. and M.C.C. drafted and revised the manuscript; J.H.C.H., S.C.C. and D.Y.C. reviewed and edited the manuscript.

## Supporting information

Supporting Information

## Data Availability

The data that support the findings of this study are available from the corresponding author upon reasonable request.
