# Supplementary material for: Targeting Dual Immune Checkpoints PD‐L1 and HLA‐G by Trispecific T Cell Engager for Treating Heterogeneous Lung Cancer
Source: Adv Sci (Weinh). 2024 Sep 5;11(41):2309697. doi: 10.1002/advs.202309697 (PMC11538689; doi:10.1002/advs.202309697)
Supplement: Supplementary file 1 — Supporting Information [file ADVS-11-2309697-s001.docx]

**Targeting Dual Immune Checkpoints PD-L1 and HLA-G by Trispecific T cell Engager for Treating Heterogeneous Lung Cancer**

Yu-Chuan Lin^#^, Mei-Chih Chen^#^, Shi-Wei Huang, Yeh Chen, Jennifer Hui-Chun Ho, Fang-Yu Lin, Xiao-Tong Tan, Hung-Che Chiang, Chiu-Ching Huang, Chih‑Yen Tu, Der-Yang Cho^*^ and Shao-Chih Chiu^*^

**Supplementary Information**

**
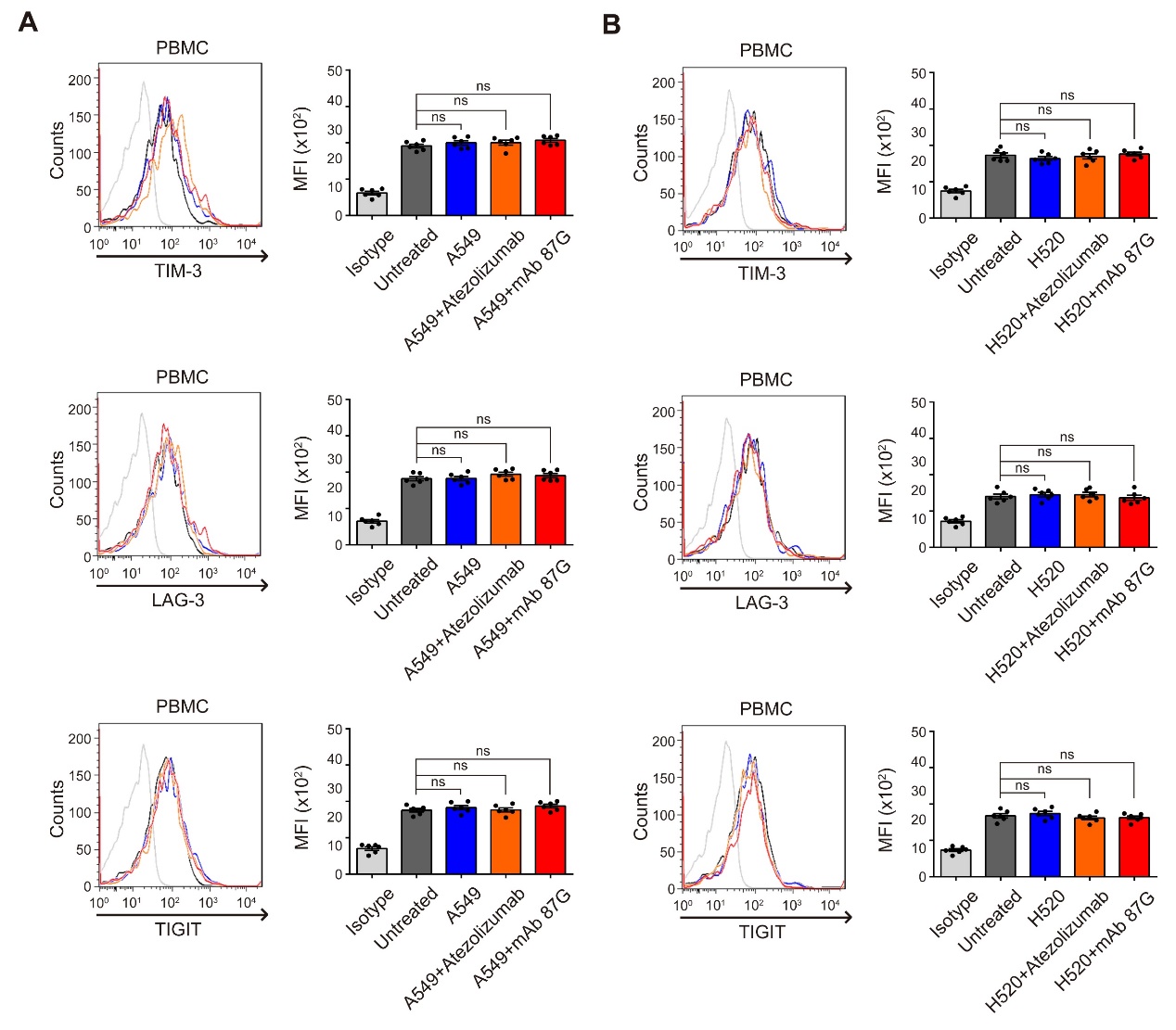
**

**Figure S1.** Assessment of the exhausted markers on PMBCs after ICB therapy. **(A)** PBMCs at 1.2 x 10^5^ cells were incubated with 4 x 10^4^ A549 cells (E:T=3:1), A549 + Atezolizumab , or A549 + mAb 87G for 24 h at 37^o^C. **(B)** PBMCs at 1.2 x 10^5^ cells were incubated with 4 x 10^4^ H520 cells (E:T=3:1), H520 + Atezolizumab, or H520 + mAb 87G for 24 h at 37^o^C. The expression of TIM-3, LAG-3, and TIGIT on the membrane of T cells was then examined by flow cytometric analysis. Quantitative results are shown as MFI ± SEM of six independent samples for each group. ns, not significant.


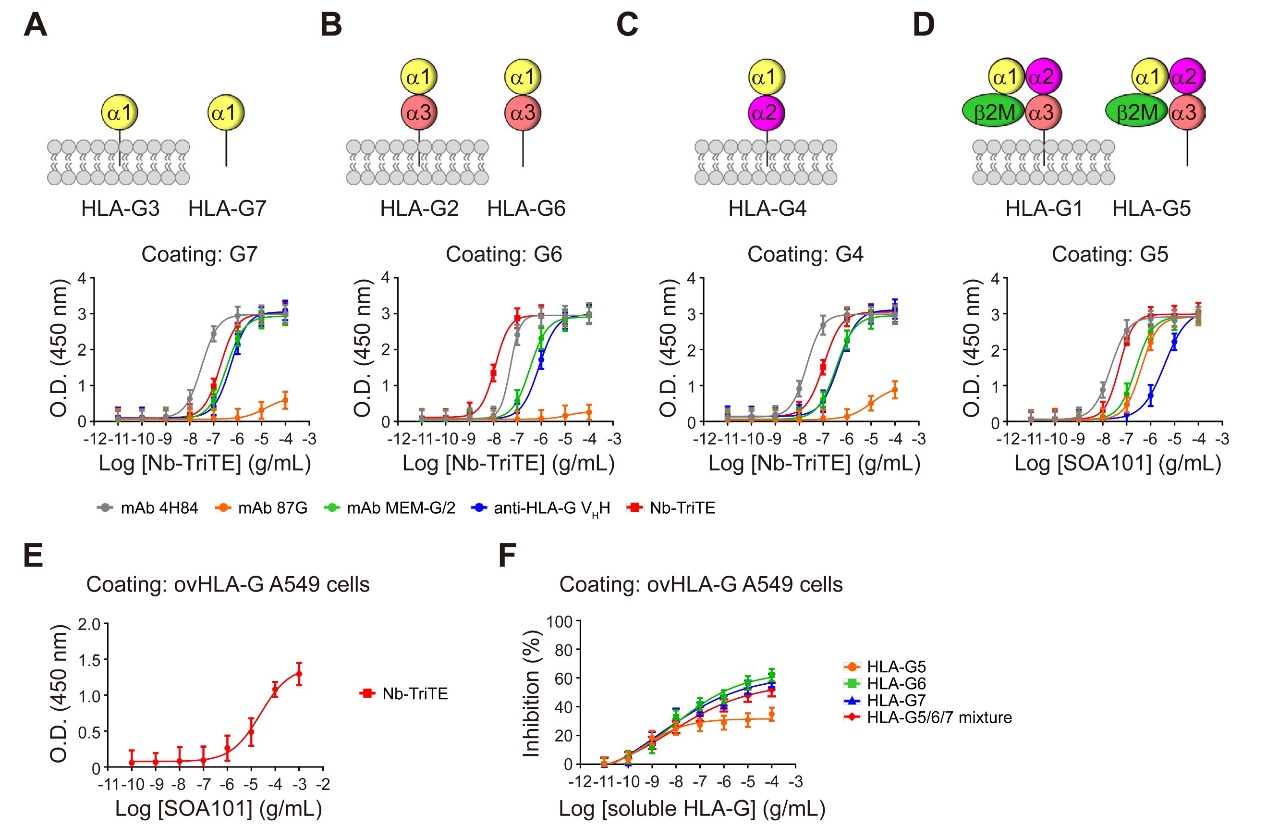


**Figure S2.** Nb-TriTE binding to HLA-G isoforms. Recombinant HLA-G isoform proteins at 0.5 mg/mL were coated on 96-well plates. The Nb-TriTE, anti-HLA-G V_H_H, and monoclonal anti-HLA-G antibodies, including mAb 4H84, mAb 87G, and mAb MEM-G/2, were then added into the 96-well plate respectively. The binding activities of Nb-TriTE and control antibodies against HLA-G isoforms, **(A)** G3 and G7 (containing α1 domain), **(B)** G2 and G6 (α1 and α3 domain), **(C)** G4 (α1 and α2 domain), or **(D)** G1 and G5 (α1, α2, and α3 domain noncovalently bound to β2-microglobulin) were evaluated by ELISA. **(E)** A549 cells overexpressing HLA-G (ovHLA-G A549 cells) at 6 x 10^3^/well was coated in 96-well plates, followed by incubated with different concentrations of Nb-TriTE. The saturated Nb-TriTE bind to 6 x 10^3^ was determined (<1000 µg/mL). **(F)** ovHLA-G A549 cells at 6 x 10^3^/well was coated in 96-well plates. In parallel, Nb-TriTE at 10 µg/mL was incubated with different concentrations of soluble HLA-G5, HLA-G6, HLA-G7, or HLA-G5/6/7 mixture. The competitive effect among Nb-TriTE and soluble HLA-G was determined using ELISA. Data represented are the means ± SEM of six independent experiments.


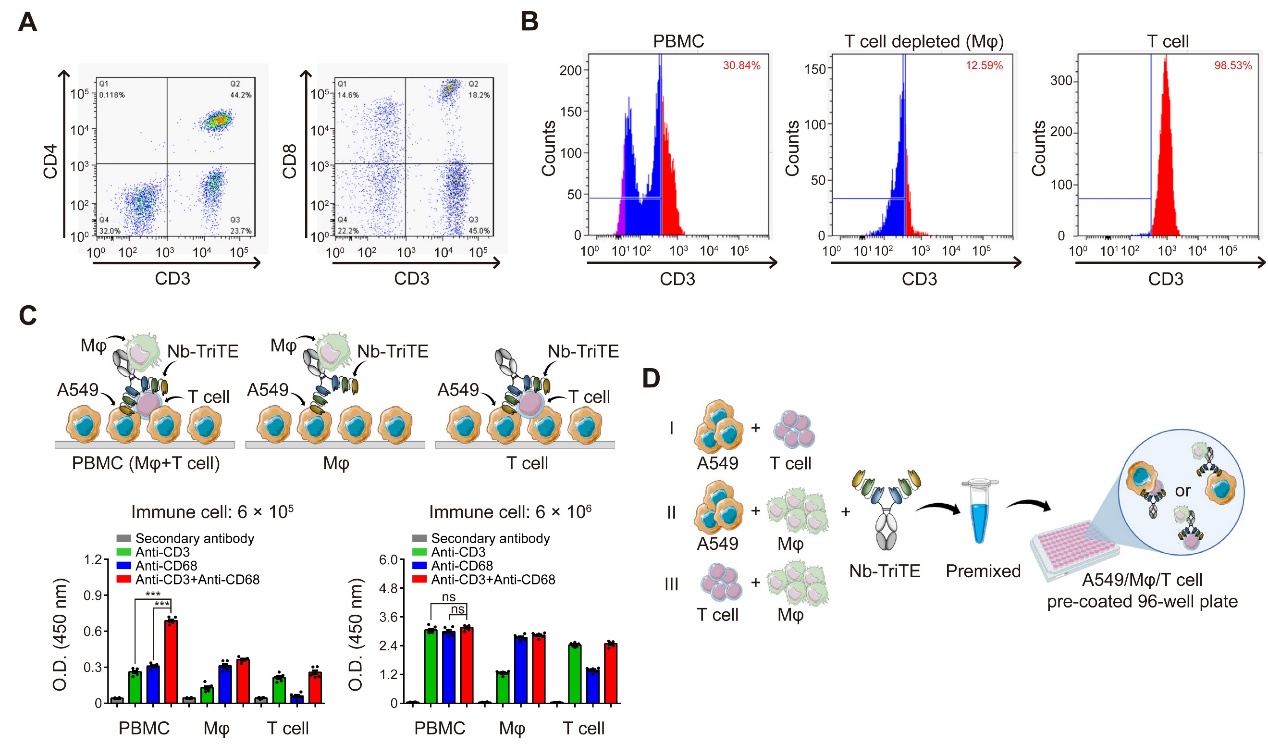


**Figure S3.** Functional assay establishment. **(A)** The percentage of CD4^+^/CD3^+^ or CD8^+^/CD3^+^ T cells in PBMCs was determined using flow cytometric analysis. **(B)** The CD3 positive T cell population in PBMCs, T cell depleted PBMCs, or isolated T cells were identified using flow cytometry. **(C)** ELISA-based functional assay was performed to evaluate the binding ability of Nb-TriTE and immune cell complex to precoated A549 tumor cells. The saturated immune cell number at 6 × 10^6^ toward to 6 × 10^3^ A549 tumor cells per well was determined. Data represent the mean ± SEM of six independent experiments. ****p*<0.001. ns, not significant. **(D)** The schematic diagram shows the experimental strategy for evaluating the binding ability among A549 cells, PBMCs and T cells in the presence of Nb-TriTE. Nb-TriTE, tumor cells and/or immune cells as illustrated in I, II and III were mixed and preincubated prior to be added into the 96-well plates. The binding affinity of Nb-TriTE and/or tumor cells/immune cells mixture to the cells that precoated on the plates was determined using ELISA. Data represented are the means ± SEM of six independent experiments. Mφ, macrophage.

**
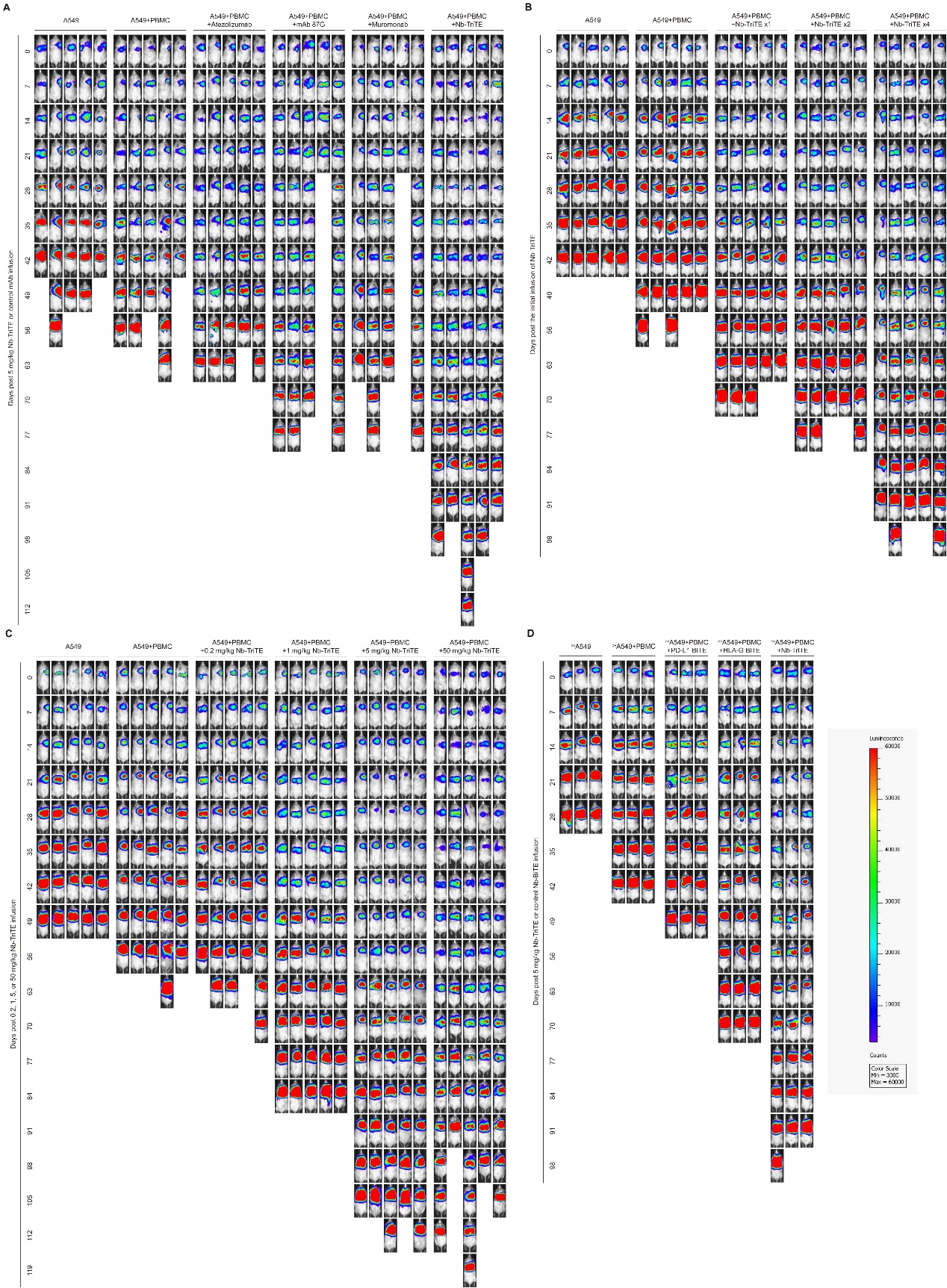
**

**Figure S4.** Nb-TriTE significantly attenuates tumor growth in A549-implanted humanized mice. Representative *in vivo* imaging system (IVIS) images were obtained weekly for comparing **(A)** the anti-cancer efficacy of Nb-TriTE and commercial monoclonal antibodies, **(B)** dosage-regimen, **(C)** dosage-ranging, and **(D)** immune checkpoint (ICP) heterogenicity of tumor cells. Mice were considered dead when the bioluminescence reached 1.5 × 10^7^. ovA549, A549 cells overexpressing both PD-L1 and HLA-G.

**
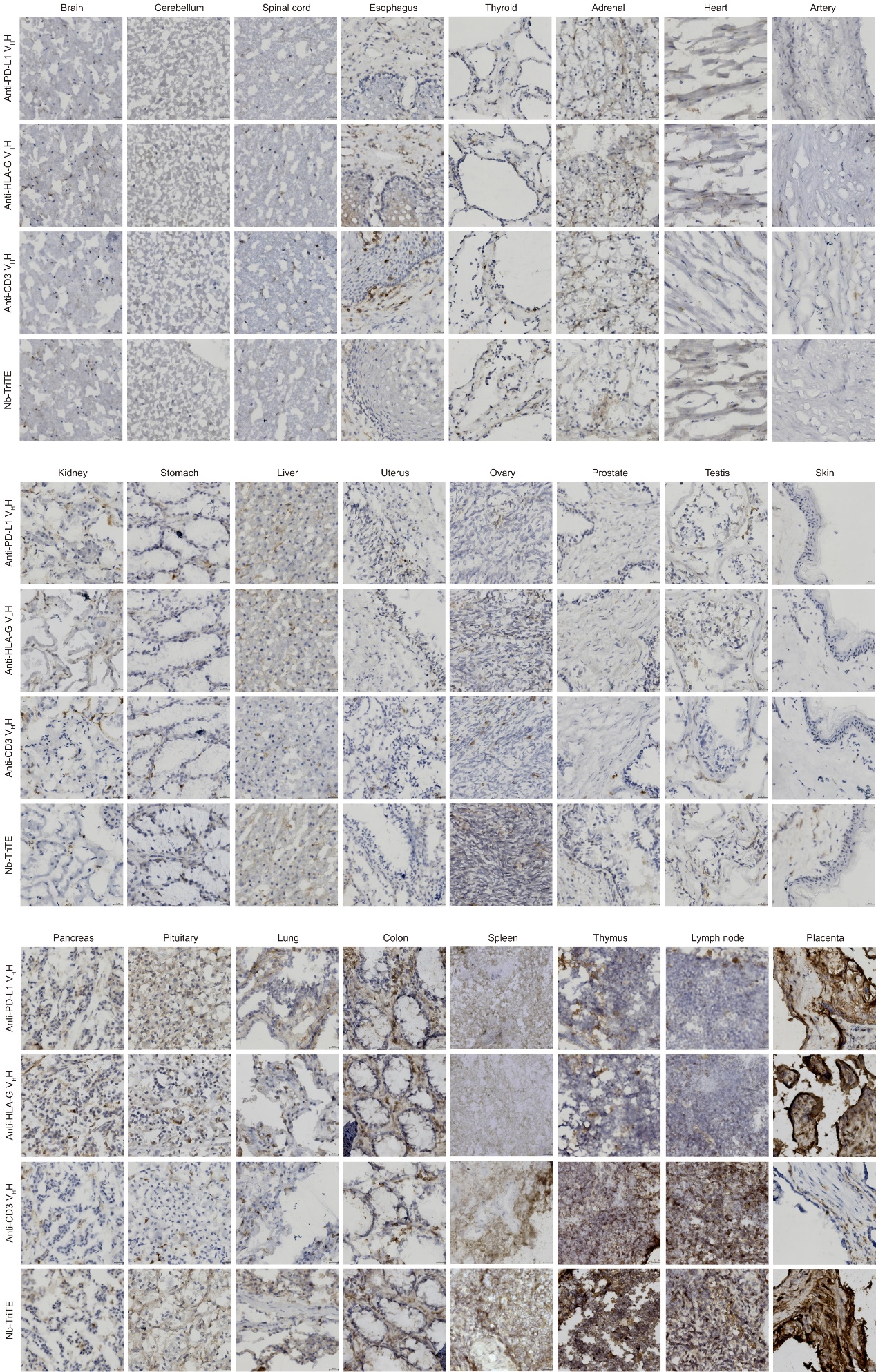
**

**Figure S5.** Tissue cross-reactivity (TCR) study for Nb-TriTE. The reactivity of anti-PD-L1 V_H_H, anti-HLA-G V_H_H, anti-CD3 V_H_H, and Nb-TriTE to 24 human normal tissues was evaluated using IHC staining. Evaluable human samples including brain, cerebellum, spinal cord, esophagus, thyroid, adrenal, heart, artery, kidney, stomach, liver, uterus, ovary, prostate, testis, skin, pancreas, pituitary, lung, colon, spleen, thymus, lymph node and placenta. Negative stain was shown in most of the human normal tissues when incubated with Nb-TriTE or the control antibodies. Nb-TriTE had relatively strong binding reactivity in placenta, lymph node, thymus, moderate reactivity in spleen and colon, and minimal reactivity in lung, pituitary, and pancreas.


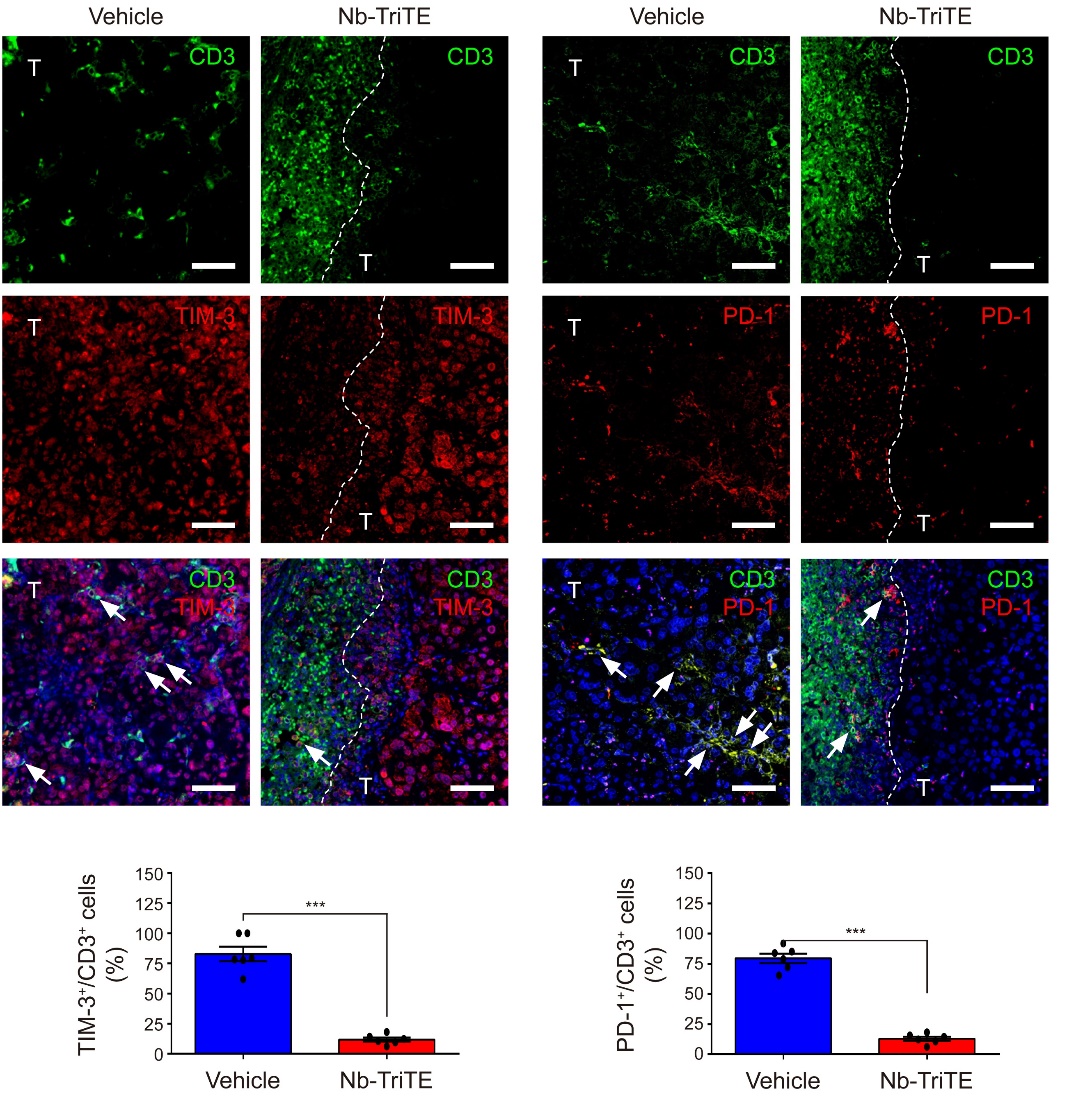


**Figure S6.** Nb-TriTE effectively enhances the recruitment of TILs and maintains their activity in the TME. The proportions of CD3^+^/TIM-3^+^ or /CD3^+^/PD-1^+^ cells in tissue sections from PBMC-humanized NSG mice bearing A549 cells with six doses of Nb-TriTE treatment were evaluated using fluorescence immunohistochemistry analysis. DAPI was used for nuclear counterstain. The dashed lines delineate the tumor margin; white arrows indicate CD3/TIM-3 or CD3/PD-1 double positive cells. The original magnification is ×400. The scale bar represents 50 μm. Quantitative results are presented as means ± SEM of six independent region of interest for each group. ***p<0.001. T, tumor region.


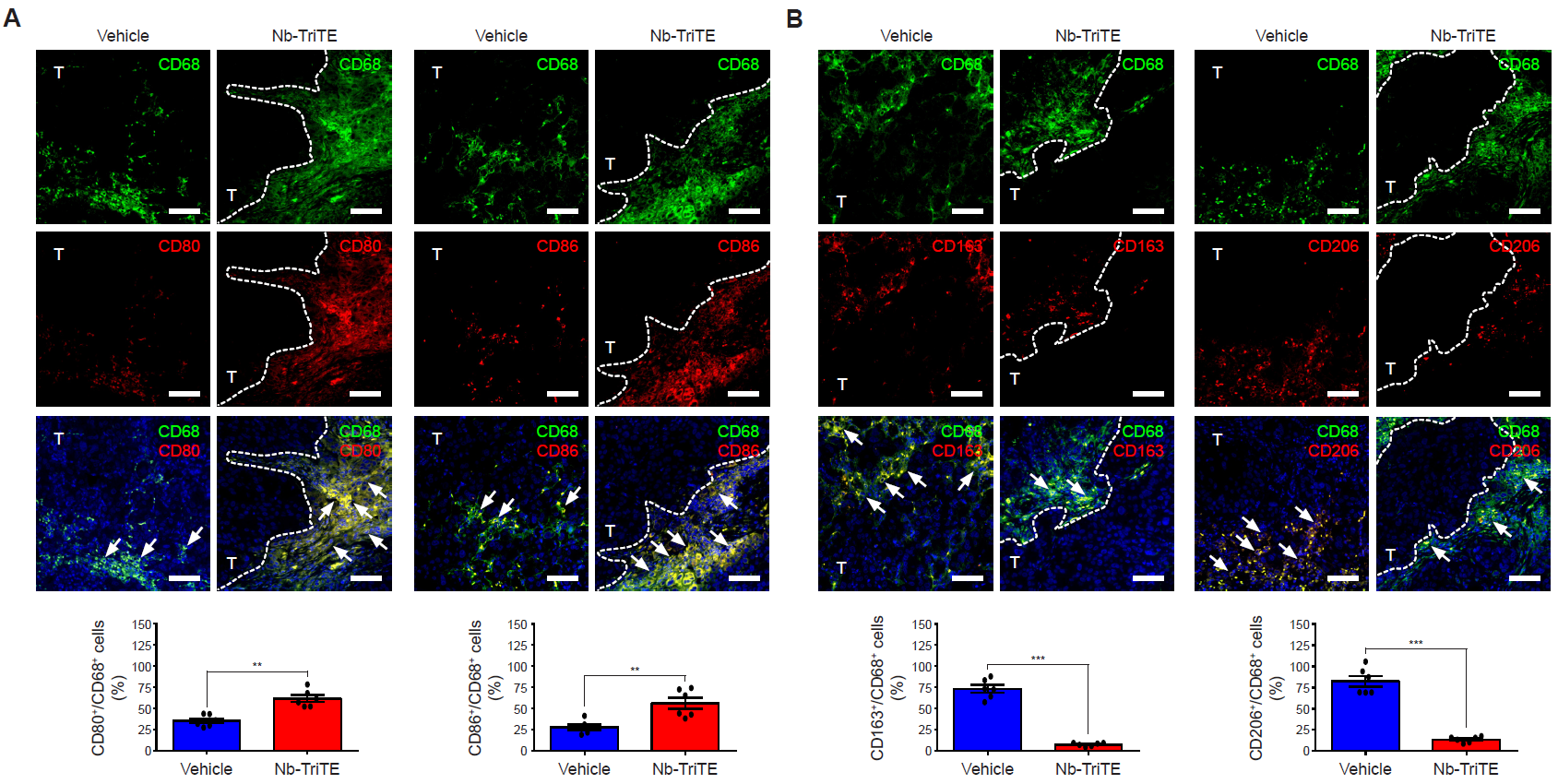


**Figure S7.** TME assessment in humanized NSG mice after treated with Nb-TriTE using fluorescence immunohistochemistry staining. The proportions of **(A)** activated M1 macrophages, such as CD68^+^/CD80^+^ and CD68^+^/CD86^+^, or **(B)** inhibitory M2 macrophages, CD68^+^/CD163^+^ and CD68^+^/CD206^+^ in tissue sections from PBMC-humanized NSG mice bearing A549 cells with six doses of Nb-TriTE treatment were evaluated using fluorescence immunohistochemistry staining. DAPI was used for nuclear counterstain. Dashed lines delineate the tumor margin; white arrows indicate CD68^+^/CD80^+^, CD68^+^/CD86^+^, CD68^+^/CD163^+^, or CD68^+^/CD206^+^ double positive cells. The original magnification is ×400. The scale bar represents 50 μm. Quantitative results are presented as means ± SEM of six independent region of interest for each group. **p<0.01; ***p<0.001. T, tumor region.


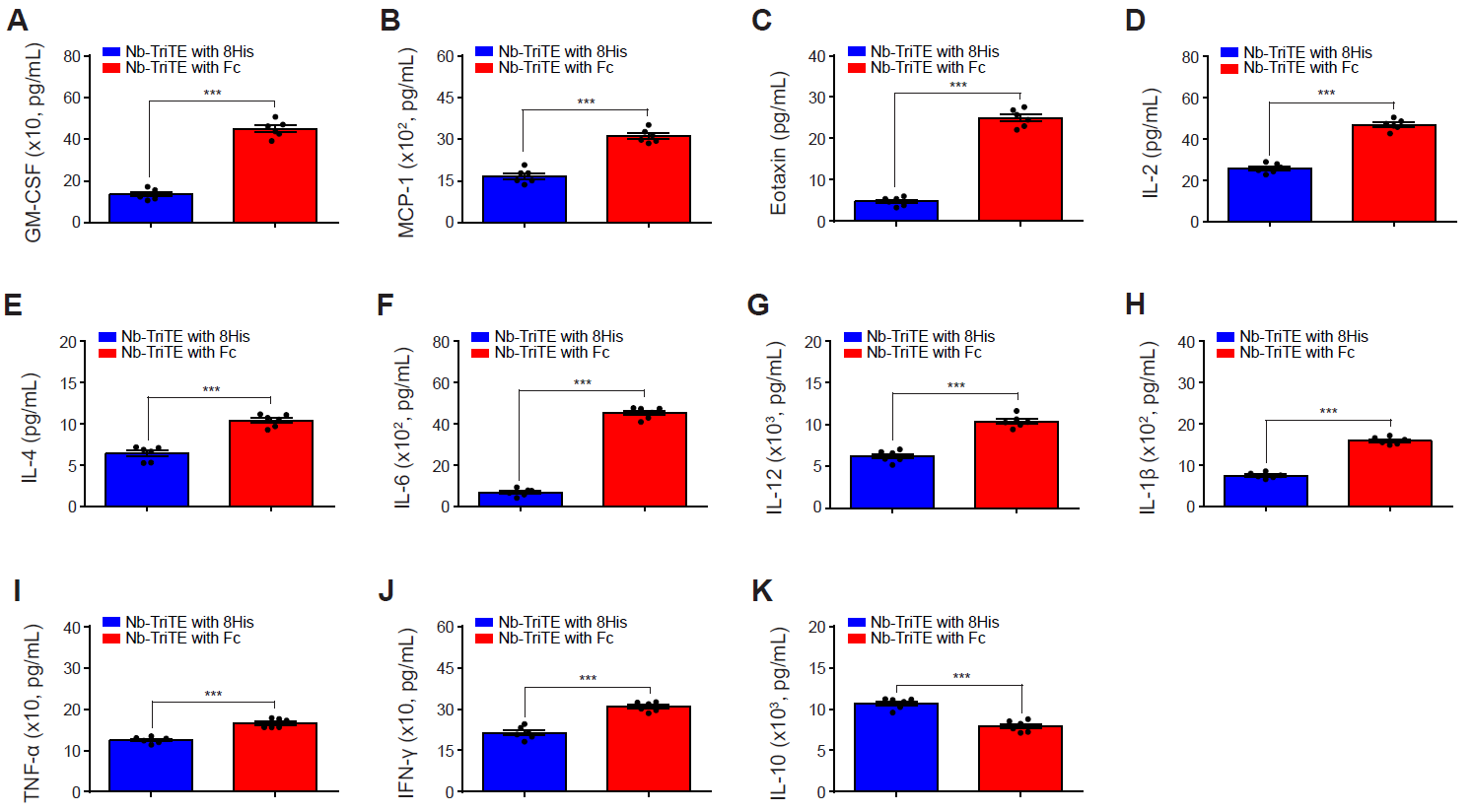


**Figure S8.** The alteration of cytokine production from PBMCs induced by Nb-TriTE with or without hIgG1 Fc-conjugation. Cytokine production from 3 x 10^4^ PBMCs coculturing with 1 x 10^4^ A549 cells (E:T= 3:1) were subjected to treat 10 µg/mL Nb-TriTE which is constructed with Fc hinge (Nb-TriTE with Fc) and a vehicle Nb-TriTE without Fc (Nb-TriTE with 8His which features 8 repeated histidine residues, served as a control to the IgG1 Fc hinge) for 24 h at 37°C. Supernatants from cell cultures were collected and analyzed by Bio-Plex Pro human cytokine assay. The release of immune activation cytokines, including **(A)** GM-CSF, **(B)** MCP-1, **(C)** eotaxin, **(D)** IL-2, **(E)** IL-4, **(F)** IL-6, **(G)** IL-12, **(H)** IL-1β, **(I)** TNF-α, and **(J)** IFN-γ were assessed as well as the immune inhibitory cytokines, **(K)** IL-10 was evaluated. Quantitative results are presented as means ± SEM of six independent samples for each group. ****p*<0.001.
